# Context-Dependent Regulation of Conjunctival Goblet Cell Function by Allergic Mediators

**DOI:** 10.1038/s41598-018-30002-x

**Published:** 2018-08-15

**Authors:** Laura García-Posadas, Robin R. Hodges, Yolanda Diebold, Darlene A. Dartt

**Affiliations:** 1 Schepens Eye Research Institute/Massachusetts Eye and Ear, Harvard Medical School, Boston, USA; 2 Department of Ophthalmology, Harvard Medical School, Boston, USA; 3 Ocular Surface Group, Institute for Applied Ophthalmobiology (IOBA), University of Valladolid, Valladolid, Spain; 4 Networking Research Center on Bioengineering, Biomaterials and Nanomedicine (CIBER-BBN), Valladolid, Spain

## Abstract

In the eye, goblet cells responsible for secreting mucins are found in the conjunctiva. When mucin production is not tightly regulated several ocular surface disorders may occur. In this study, the effect of the T helper (Th) 2-type cytokines IL4, IL5, and IL13 on conjunctival goblet cell function was explored. Goblet cells from rat conjunctiva were cultured and characterized. The presence of cytokine receptors was confirmed by Reverse Transcription-Polymerase Chain Reaction (RT-PCR). Changes in intracellular [Ca^2+^], high molecular weight glycoconjugate secretion, and proliferation were measured after stimulation with Th2 cytokines with or without the allergic mediator histamine. We found that IL4 and IL13 enhance cell proliferation and, along with histamine, stimulate goblet cell secretion. We conclude that the high levels of IL4, IL5, and IL13 that characterize allergic conjunctivitis could be the reason for higher numbers of goblet cells and mucin overproduction found in this condition.

## Introduction

Goblet cells are specialized cells that produce and secrete mucins that lubricate and protect mucosal tissues, maintaining their health^1,2^. In the ocular surface, goblet cells are found in the epithelial layer of the conjunctiva. One of the most important mucins secreted by conjunctival goblet cells is MUC5AC, a high molecular weight glycoconjugate that forms the mucous layer of the tear film^3^. The amount of MUC5AC found in the ocular surface is tightly controlled by goblet cell number, MUC5AC synthesis, and MUC5AC secretion. Either an increase or a decrease in the number of secretory granule-filled goblet cells is associated with ocular surface pathology^4^.

Under normal conditions, goblet cell secretion is under neural control by the efferent, parasympathetic nervous system. Cholinergic, muscarinic agonists that are derivatives of the parasympathetic neurotransmitter acetylcholine are major stimuli^5^. However, when inflammation is present, the signaling pathways that mediate goblet cell secretion can change.

Inflammation is a pathogenic mechanism underlying numerous ocular disorders. One important inflammatory mediator present in allergy is histamine that in the conjunctiva is secreted by mast cells recruited into the stroma^6^. Histamine causes an inflammatory response characterized by vasodilation and increased vascular permeability^7^.

Histamine acts by binding to its receptor H, of which there are four. On conjunctival goblet cells all four histamine receptors are present and histamine activates all four of the histamine receptors H_1_-H_4_^8^. For H_1_, H_3_, and H_4_ histamine activates phospholipase C to release [Ca^2+^]_i_ that induces the influx of extracellular Ca^2+^. This influx activates ERK1/2 to stimulate conjunctival goblet cell secretion^9^. For H_2_ histamine increases cAMP that increases [Ca^2+^]_i_^10^.

There is a growing interest in determining the role played by cytokines in the inflamed conjunctiva. There are different types of cytokines, and a way to classify them depends on the type of cell that predominantly secretes them. One of the major sources of cytokines is T helper (Th) lymphocytes. These Th cells can be divided in different subsets, such as Th1, Th2, Th17, or the most recently described Th22-type, and the cytokines are similarly divided. The most widely studied and clearly defined cytokines are the Th1 and Th2-types^11,12^. Th1 cytokines are involved in the pathogenesis of some autoimmune diseases^13^. We recently showed the role of interferon gamma (IFN-γ), the major Th1 cytokine, in goblet cell function modulation^14^.

Interleukin (IL) 4, IL5, and IL13 are Th2 cytokines, and are implicated in allergic processes. They are responsible for IgE production by B cells, mast cell growth, eosinophil accumulation and mucus hyperproduction^15^. Each cytokine has a specific receptor (R). IL4-R is composed of two subunits: an α subunit (which is also a component of IL13-R) and a γc subunit that amplifies signaling of IL4-Rα^16^. IL5-R is comprised of an IL5-Rα specific subunit and a common β subunit^17^. IL13-R is composed of IL4-Rα chain and IL13Rα1 chains^16^. A role for these cytokines in ocular allergy has been widely demonstrated^18–20^. However, as in the case of IFN-γ, the early effects of these cytokines on conjunctival goblet cell regulation have not been studied yet.

The purpose of this study is to determine whether IL4, IL5 and IL13, players in allergic conjunctivitis, directly regulate mucin production in rat conjunctival goblet cells. To this end presence of cytokine receptors, as well as effect on intracellular Ca^2+^ signaling, mucin secretion, and goblet cell proliferation, was measured after stimulation by the cytokines alone or in the presence of a mediator of allergy, histamine. We found that the Th2 cytokines increased goblet cell proliferation, but blocked histamine-stimulated increase in [Ca^2+^]_i_ and mucin secretion. Thus, Th2 cytokines lead to increased goblet cell number.

## Results

### Goblet Cell Culture and Characterization

**Figure 1:**
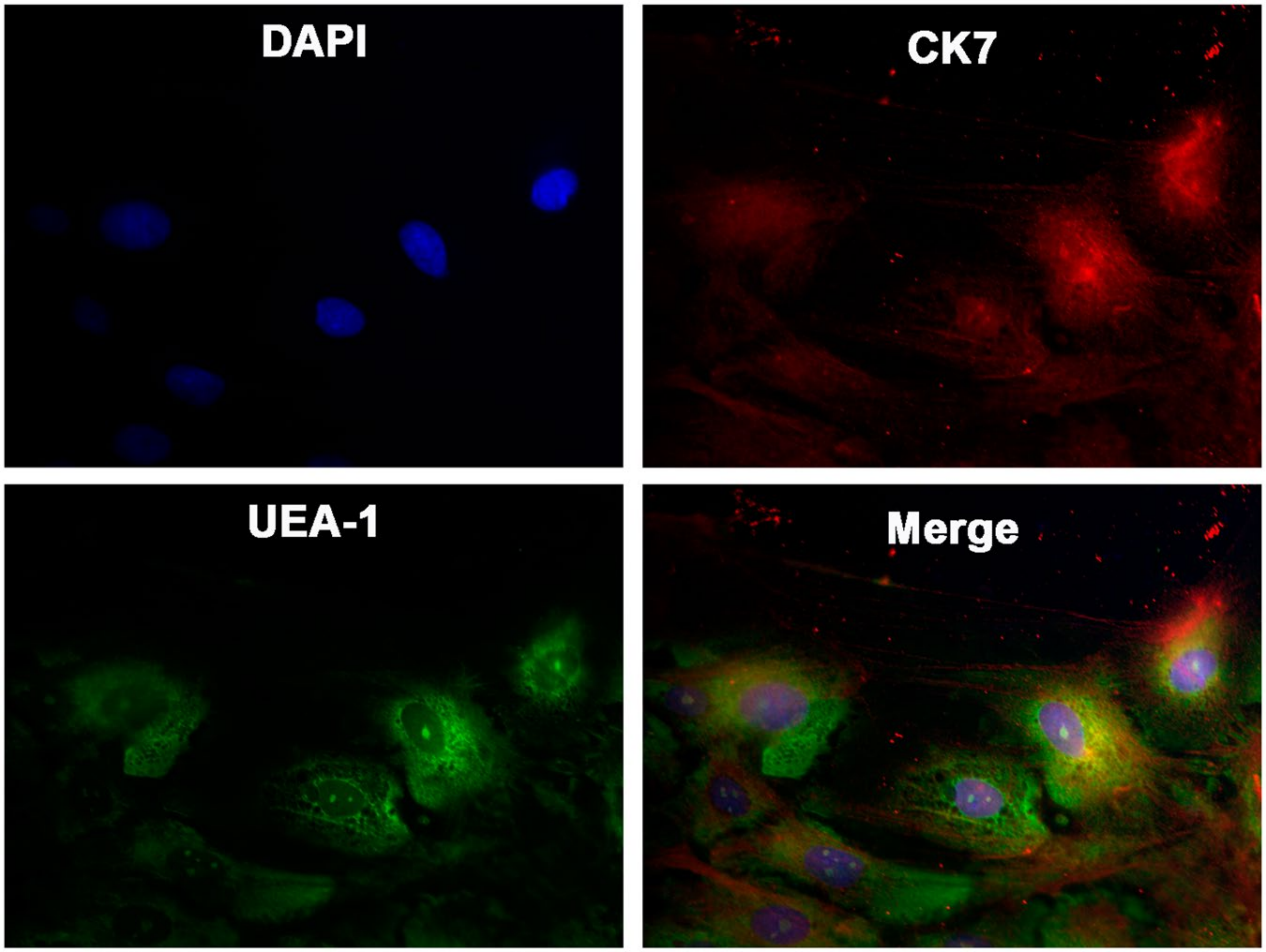
Representative images of rat cultured conjunctival goblet cells. Nuclei were stained in blue with DAPI. CK7 localization is indicated in red and lectin UEA-1 in green. Magnification 400x.

Identity of cultured cells was confirmed by immunofluorescence microscopy using the following markers: cytokeratin (CK) 7 (goblet cell specific keratin)^21,22^, and lectin *Ulex europaeus agglutinin* type 1 (UEA-1). Lectins identify high molecular weight glycoconjugates including MUC5AC synthesized and secreted by goblet cells. Almost all of the cells in culture were positive for both CK7 (red) and lectin UEA-1 (green) as shown in Fig. 1. Therefore, the overwhelming majority of cells in culture were goblet cells.

### Goblet cells express IL4-R, IL5-RA, and IL13-RA1

**Figure 2:**
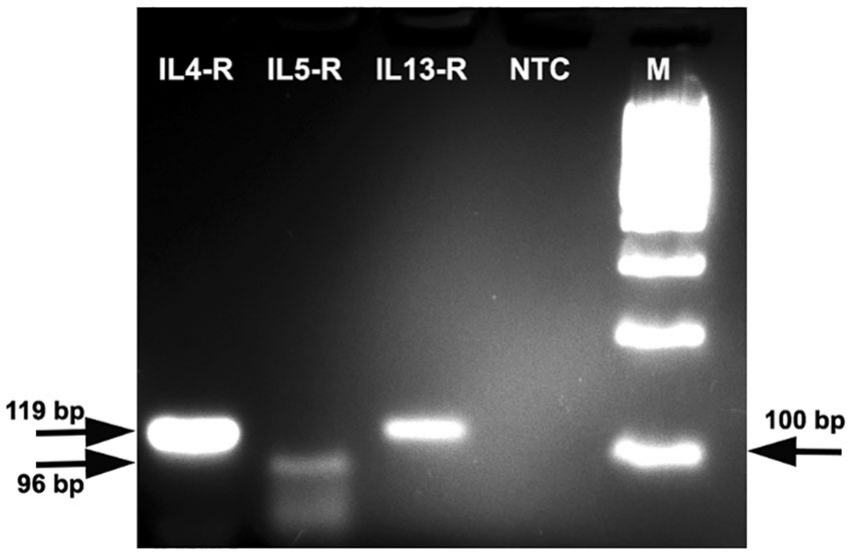
Presence of MUC5AC and cytokine receptors in rat conjunctival goblet cells shown by RT-PCR. M = base pair marker; NTC = non template control; R = receptor; arrows indicate expected base pairs for each receptor.

To determine if conjunctival goblet cells were able to directly respond to the selected cytokines, receptors (R) for those cytokines were studied by Reverse Transcription-Polymerase Chain Reaction (RT-PCR). In rat IL4-R (119 bp), IL5-RA (96 bp), and IL13-RA1 (119 bp) mRNAs were detected (Fig. 2). We observed two bands in IL5-RA. It has been described that IL5-RA shows alternative splicing to give two different variants^23^, which could explain the two bands observed in the agarose gel. We confirmed that rat cultured cells expressed all the receptors needed for IL4, IL5, and IL13 to be effective.

### IL4, IL5 and IL13 increase [Ca^2+^]_i_ in rat goblet cells

Due to its role in goblet cell signaling, the intracellular calcium level ([Ca^2+^]_i_) was measured in cultured cells after addition of different stimuli. To determine the effect of Th2-type cytokines, the inflammatory mediator histamine was used as a compound that was representative of allergic inflammation-mediated response. Histamine known to increase [Ca^2+^]_i_ in conjunctival goblet cells was used as the positive control. Concentration-dependency assays were first performed to select optimal concentrations for controls and cytokines (data not shown).

**Figure 3:**
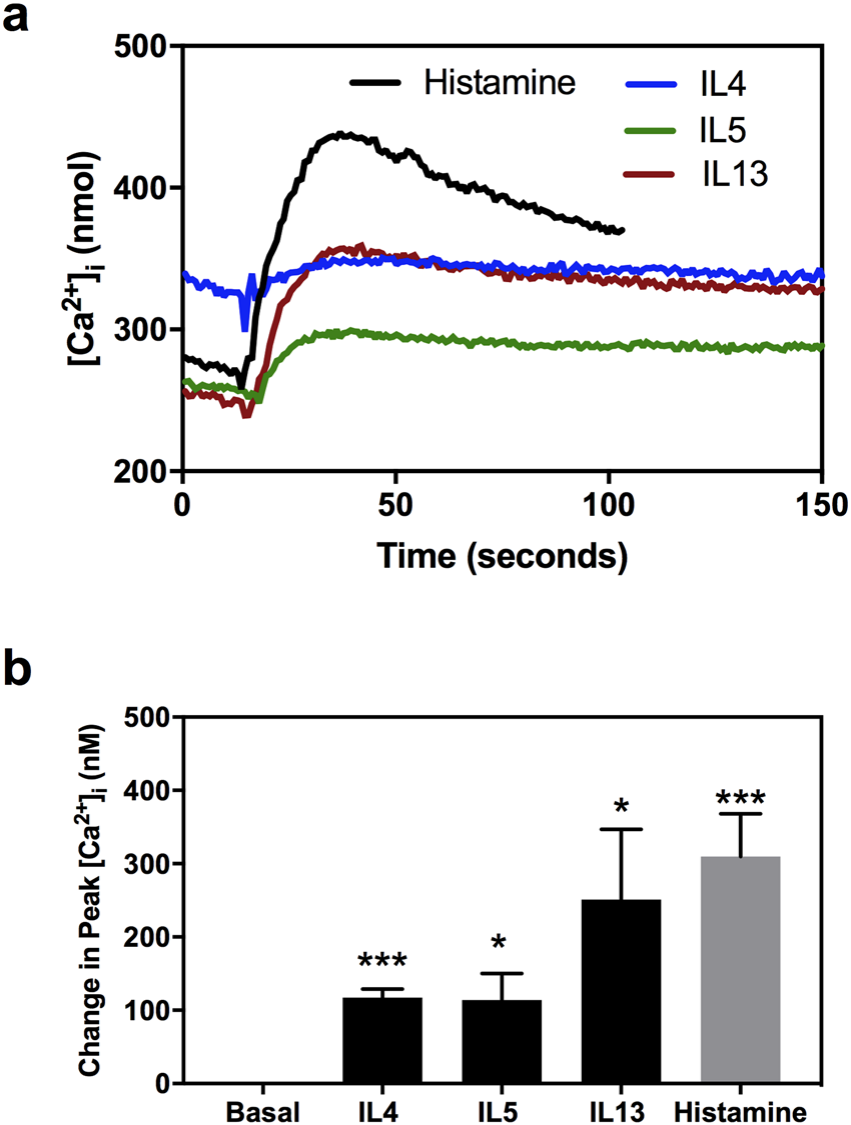
Histamine and Th2 cytokines increase in intracellular [Ca^2+^] ([Ca^2+^]_i_) in rat conjunctival goblet cells. (**a**) Mean [Ca^2+^]_i_ over time after addition of 10^−5^ M histamine, 10 ng/ml IL4, 10 ng/ml IL5, or 10 ng/ml IL13. (**b**) Peak value over basal in response to histamine, IL4, IL5 or IL13. Data are expressed as mean ± SEM of four independent experiments. ^*^indicates statistical significance compared to basal values. ^*^p ≤ 0.05; ^***^p ≤ 0.005.

The Th2 cytokines IL4, IL5, and IL13 significantly increased peak [Ca^2+^]_i_ to 117.16 ± 11.94 nM (p = 0.0006), 114.02 ± 36.27 nM (p = 0.020), and 251.17 ± 95.89 nM (p = 0.039), respectively (Fig. 3). Histamine, the positive control, significantly increased peak [Ca^2+^]_i_ to 309.18 ± 58.60 nM (p = 0.002), as expected. The effects of IL4 and IL5 were significantly lower than that of histamine (p = 0.018 and p = 0.030, respectively). In summary, all Th2 cytokines studied increased peak [Ca^2+^]_i_ in conjunctival goblet cells, but only IL13 did it in levels similar to histamine.

### Th2 cytokines block histamine-induced increase in [Ca^2+^]_i_ at different times of pre-incubation

**Figure 4:**
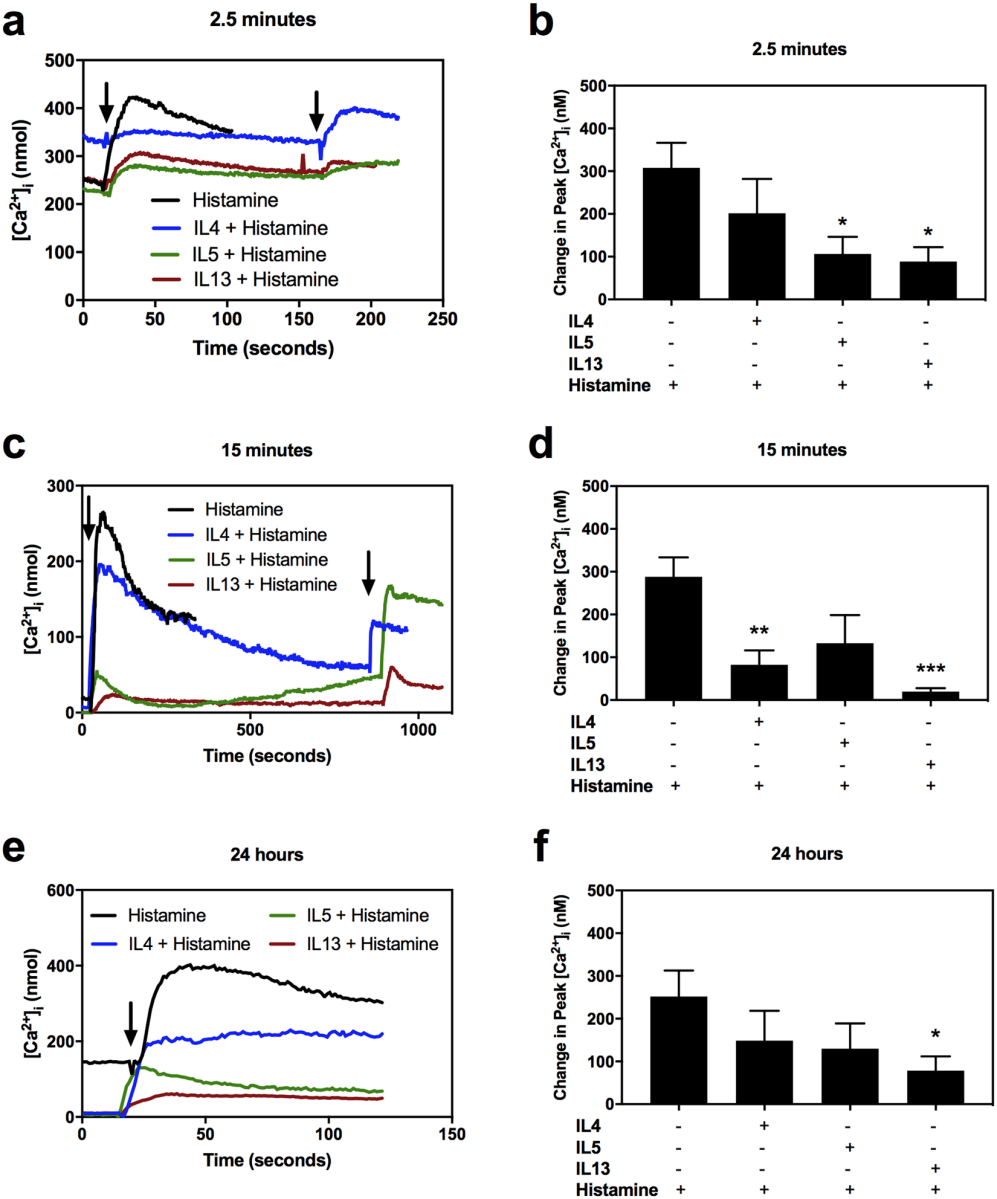
Effect of IL incubation time on histamine stimulated increase in intracellular [Ca^2+^] ([Ca^2+^]_i_) in rat conjunctival goblet cells. Mean [Ca^2+^]_i_ response to histamine over time (**a**,**c**,**e**) or peak [Ca^2+^]_i_ over basal (**b**,**d**,**f**) after treatment with cytokines at 10 ng/ml each (first arrow) for 2.5 min (**a**,**b**), 15 min (**c**,**d**), or 24 h (**e**,**f**) before the addition of 10^−5^ M histamine (second arrow). At least three independent experiments were performed for each condition. * indicates statistical significance compared to histamine alone. ^*^p ≤ 0.05; ^**^p ≤ 0.01; ^***^p ≤ 0.005.

To determine the effect of the cytokines on histamine mediated increase in [Ca^2+^]_i_, three different cytokine preincubation times were selected: 2.5 min, 15 min, and 24 h, to study immediate, short, and long-term responses, respectively. The 2.5 min pre-treatment blocked the histamine-induced response with IL5 and IL13, from 307.75 ± 83.24 nM to 106.51 ± 48.89 nM (p = 0.048) and to 88.44 ± 41.58 nM (p = 0.033), respectively (Fig. 4a,b). IL4 did not block the histamine response.

The 15 min pre-treatment with these cytokines revealed a blockade in histamine-induced response, from 288.18 ± 45.29 nM to 82.39 ± 33.58 nM with IL4 treatment (p = 0.006) and to 19.61 ± 7.985 nM (p = 0.003) with IL13 treatment (Fig. 4c,d). IL5 did not diminish the histamine response.

The longest pre-treatment (24 h) did not show a significant blockade with IL4 or IL5, but the treatment with IL13 diminished the effect of histamine on [Ca^2+^]_i_ from 252.00 ± 60.9 nM to 78.45 ± 33.43 nM (Fig. 4e,f).

In summary, the effect of the cytokines on histamine response is time dependent. Only IL13 blocked histamine-mediated increase in [Ca^2+^]_i_ at all times studied.

### Cytokine effect on histamine is predominantly produced through H1 receptor

To better analyze the signaling pathways used by cytokines and histamine to exert their effects, a histamine receptor specific agonist, histamine dimaleate, was used. Histamine dimaleate is an H1 agonist. We observed that the effect of histamine dimaleate was very similar to that of histamine, suggesting that H1 is the main H receptor in conjunctival goblet cells, as previously suggested^8,9^.

**Figure 5:**
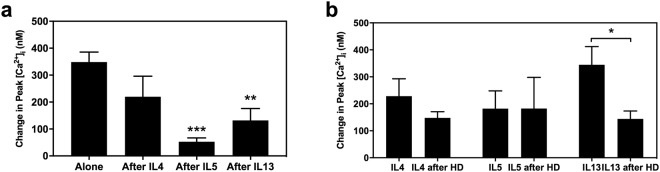
Effect of IL pre-treatment on increase in intracellular [Ca^2+^] ([Ca^2+^]_i_) stimulated by the H1 agonist histamine dimaleate (HD) in rat conjunctival goblet cells. Peak [Ca^2+^]_i_ over basal produced by the addition of 10^−5^ M HD after a pre-treatment with IL4, IL5, or IL13 each at 10 ng/ml for 2.5 min (**a**) or by the addition of IL4, IL5, or IL13 alone or after a pre-treatment with 10^−5^ M HD (**b**). Data are expressed as means ± SEM from five independent experiments. ^*^p ≤ 0.05; ^**^p ≤ 0.01; ^***^p ≤ 0.005.

IL5 and IL13 each blocked the histamine dimaleate increase in peak [Ca^2+^]_i_ after 2.5 min treatment (Fig. 5a), similarly to the effect obtained with histamine (Fig. 4a,b). In addition, histamine dimaleate blocked the IL13-mediated increase in [Ca^2+^]_i_, but not the IL4 or IL5 stimulated increase (Fig. 5b).

### Use of extracellular and intracellular calcium stores

**Figure 6:**
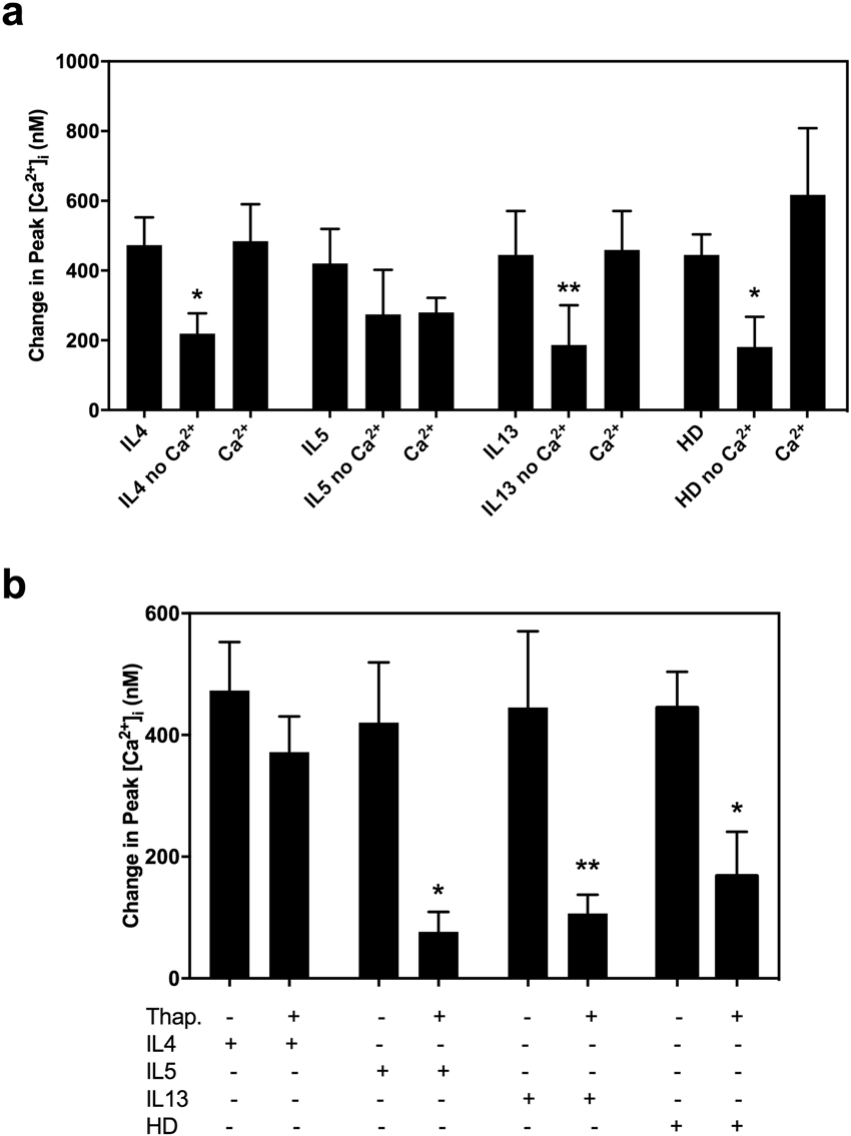
Effect of extracellular and intracellular Ca^2+^ stores on IL cytokine induced increase in intracellular [Ca^2+^] ([Ca^2+^]_i_) in rat conjunctival goblet cells. (**a**) Peak [Ca^2+^]_i_ over basal after stimulation with IL4, IL5, IL13 each at 10 ng/ml and histamine dimaleate (HD) at 10^−5^ M in the presence of extracellular Ca^2+^ or in the absence of extracellular Ca^2+^ (no Ca^2+^), and followed by the readdition of extracellular Ca^2+^. (**b**) Peak [Ca^2+^]_i_ over basal for IL4, IL5, IL13 and histamine dimaleate (HD) alone or after a 15 min treatment with 10^−5^ M thapsigargin (Thap.). Data are mean ± SEM from five independent experiments. ^*^p ≤ 0.05; ^**^p ≤ 0.01.

The calcium stores used by three cytokines were studied. To determine if the cytokines used extracellular calcium, cells were incubated in buffer without Ca^2+^, then extracellular Ca^2+^ was added back (Fig. 6a). When extracellular Ca^2+^ was removed, the response to IL4, IL13, and the positive control histamine dimaleate was significantly reduced, indicating that these agonists used extracellular calcium. In contrast, the effect of IL5 on [Ca^2+^]_i_ did not change in the absence of extracellular calcium. When extracellular Ca^2+^ was added back, the [Ca^2+^]_I_ response to IL4, IL13, and histamine dimaleate returned to the original level and was no longer decreased.

To determine if the cytokines use intracellular calcium, cells were treated with thapsigargin, a compound that depletes endoplasmic reticulum stores (Fig. 6b). After thapsigargin treatment the response to IL5, IL13, and histamine dimaleate was significantly reduced. However, the IL4-mediated increased in [Ca^2+^]_i_ remained unaltered.

These results showed that histamine dimaleate and IL13 use both intracellular and extracellular Ca^2+^ stores, whereas IL4 uses mainly extracellular stores, and IL5 uses intracellular stores.

### IL5 uses Beta-Adrenergic Receptor Kinase 1 (β-ARK1) to counter regulate the H1 receptor

**Figure 7:**
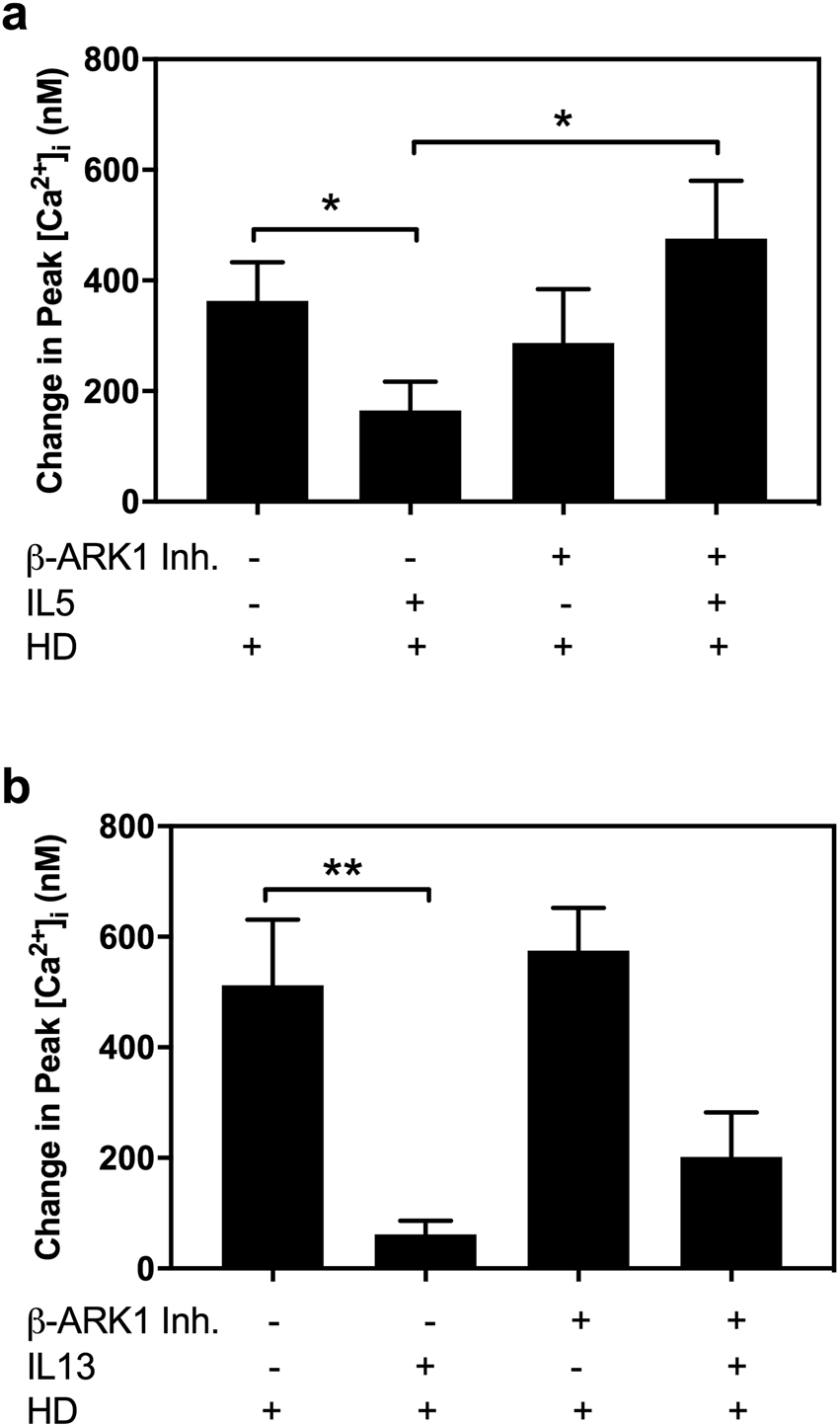
Change in peak [Ca^2+^]_i_ in rat conjunctival goblet cells. Cells were stimulated with either histamine dimaleate (HD, 10^−5^ M); preincubated with 10 ng/ml IL 5 (**a**) or 10 ng/ml IL13 (**b**) for 30 min prior to addition of the HD; or preincubated with β-adrenergic receptor kinase 1 inhibitor peptide (β-ARK1 Inh peptide, 10^−6^ M) added 30 min before HD; or preincubated with β-ARK1 Inh peptide, 10^−6^ M, for 15 min prior to addition of 10 ng/ml IL5 (**a**) or 10 ng/ml IL13 (**b**) that were added 30 min before HD. Data are mean ± SEM from eight independent experiments (**a**) or four independent experiments (**b**). ^*^≤0.05; ^**^≤ 0.01.

IL5 when incubated for 2.5 min blocked the increase in [Ca^2+^]_i_ induced by histamine dimaleate in goblet cells from 363.06 ± 69.65 nM to 164.78 ± 52.4 nM (Fig. 7a). When cells were pretreated with 10^−8^M β-ARK1 inhibitor for 30 min before the addition of IL5, the blockade of the subsequent histamine dimaleate response produced by IL5 disappeared. As a control, β-ARK1 inhibitor added for 30 min before histamine dimaleate did not alter this response.

IL13 treatment for 2.5 min blocked the increase in [Ca^2+^]_i_ induced by histamine dimaleate from 512.07 ± 119.0 nM to 61.61 ± 24.93 nM (Fig. 7b). The addition of β-ARK1 inhibitor for 30 min before addition of IL-13 did not reverse the IL-13 inhibition of the subsequent histamine dimaleate response. As a control, β-ARK1 inhibitor added for 30 min before histamine dimaleate did not alter this response. The effect of IL4 was not tested as this cytokine did not block the histamine stimulated response when treated for 2.5 min (Fig. 5a).

Therefore, IL5 uses β-ARK1 to counter regulate histamine-mediated increase in [Ca^2+^]_i,_ whereas the blockade produced by IL13 appears to be using a different mechanism.

### IL13 prevents interaction of IL4 with its receptor

**Figure 8:**
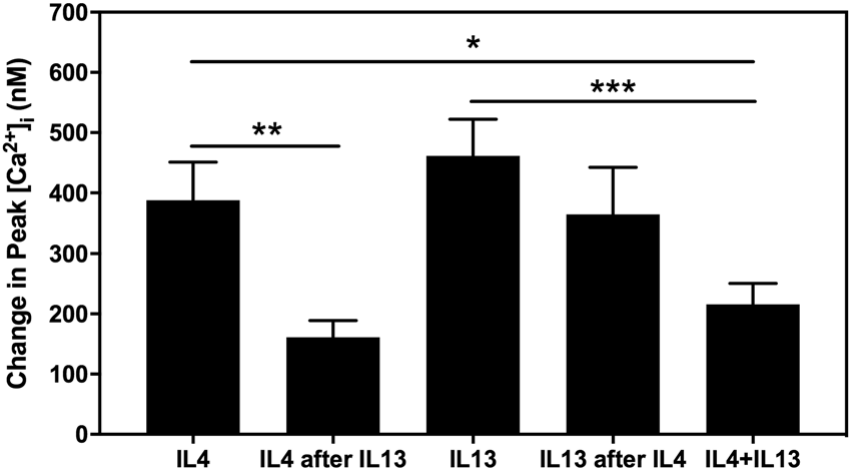
Effect of IL4 and IL13 on each other’s increase in intracellular [Ca^2+^] ([Ca^2+^]_i_) in rat conjunctival goblet cells. Change in peak [Ca^2+^]_i_ over basal produced by 10 ng/ml IL4 or IL13 alone; or on IL4 or IL13 after a 2.5 min pretreatment with IL13 or IL4, respectively; or by a combination of both IL4 and IL13 added simultaneously. Data are mean ± SEM from eight independent experiments. ^*^≤0.05; ^**^≤ 0.01; ^***^≤0.005.

Since IL4 and IL13 share a common receptor, the interaction of these cytokines was studied. IL13 treatment for 2.5 min blocked the IL4-mediated increase in [Ca^2+^]_i_ from 387.84 ± 63.27 nM to 161.35 ± 27.59 nM, whereas IL4 pre-treatment had no effect on the IL13 response (Fig. 8). When both cytokines were added together the increase in peak [Ca^2+^]_i_ was significantly lower (215.88 ± 34.63 nM) than that obtained with either IL4 (387.84 ± 63.27 nM) or IL13 (461.01 ± 60.88 nM) alone (Fig. 8). These results show that the presence of IL13 reduces the effect of IL4 in conjunctival goblet cells.

### Histamine and IL13 induce mucin secretion from cultured rat goblet cells

**Figure 9:**
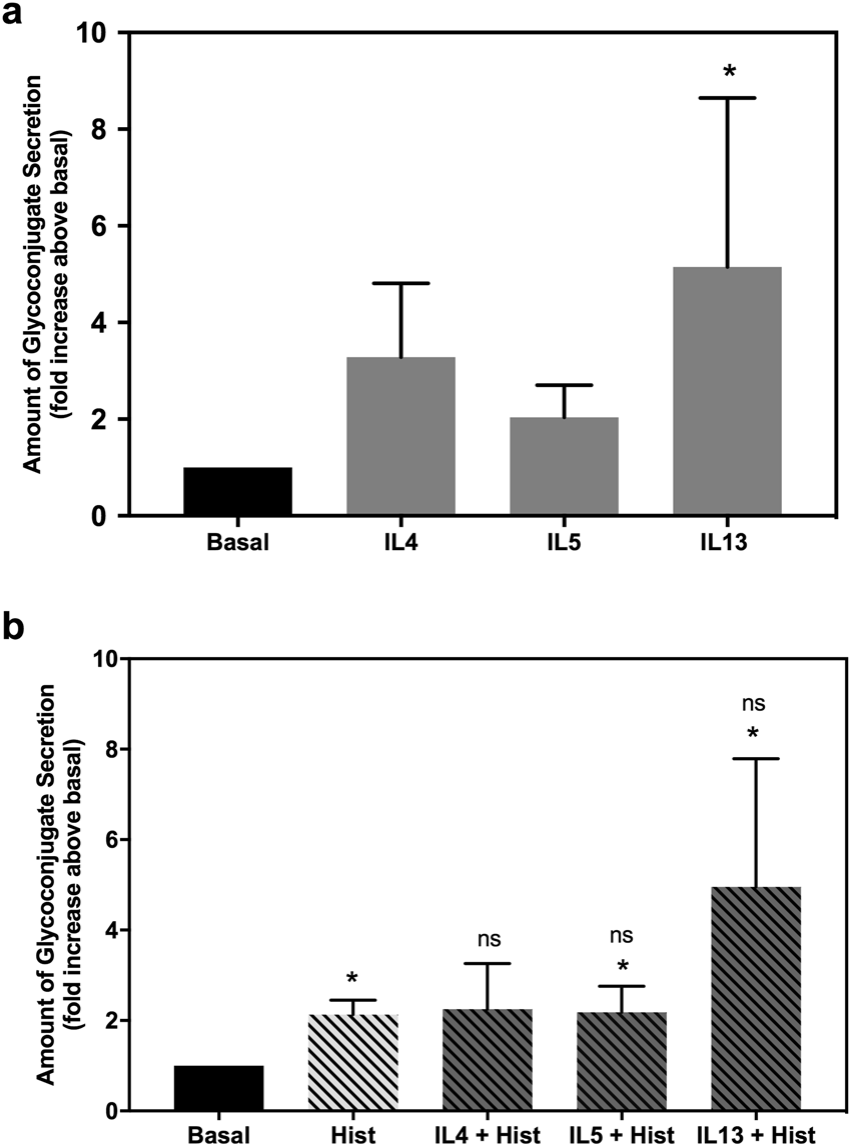
Effect of cytokines alone or on histamine stimulated rat conjunctival goblet cell secretion. **(a)** Glycoconjugate secretion stimulated with 10 ng/ml IL4, IL5, or IL13 for two hours. (**b**) Glycoconjugate secretion stimulated by 10^−5^ M histamine (Hist) alone for two h or after pretreatment for 30 min with IL4, IL5 or IL13. Data are mean ± SEM from five independent experiments. * indicates statistical significance compared to basal values; ns indicates no significance from histamine alone.

Goblet cell secretion was evaluated after 2 h stimulation with cytokines. Neither IL4 nor IL5 increased secretion, whereas IL13 increased it by 5.15 ± 3.49 fold above basal (Fig. 9a).

After 2 h stimulation, histamine induced high molecular weight glycoconjugate secretion by rat goblet cells, as previously demonstrated^9^. After stimulation with IL4, IL5, or IL13 for 30 min, histamine was added to study the effect of the individual cytokines on the secretory effect of histamine. There was no effect of any of the ILs on histamine-induced secretion (Fig. 9b).

### IL4 and IL13 increase goblet cell proliferation

**Figure 10:**
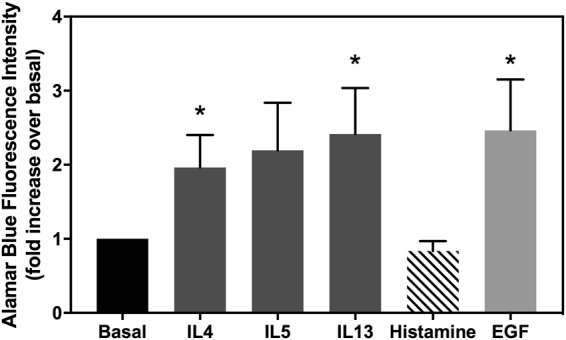
Effect of cytokines on conjunctival goblet cell proliferation. Goblet cells were incubated for 24 h with 10 ng/ml IL4, 10 ng/ml IL5, 10 ng/ml IL13, 10^−5^ M histamine, or 10^−7^ M EGF and cell proliferation measured. Data are mean ± SEM from seven independent experiments. ^*^≤0.05.

Cellular proliferation was measured in rat conjunctival goblet cells with Alamar Blue (Fig. 10). Epidermal growth factor (EGF), known to increase epithelial cell proliferation and conjunctival goblet cells in particular, was used as a positive control^24^. After 24 h treatment with histamine, the individual cytokines, or EGF, number of viable cells was studied with Alamar Blue reagent. IL4 and IL13 increased rat goblet cell proliferation (1.96 ± 0.44 fold increase, p = 0.048, and 2.41 ± 0.62 fold increase, p = 0.042, respectively), whereas histamine and IL5 did not have any significant effect. However, a tendency towards higher proliferation rates was observed for IL5 (2.19 ± 0.64 fold increase, p = 0.087). As expected, EGF induced a 2.46 ± 0.69 fold increase in cell proliferation (p = 0.041). The effects of IL4 and IL13 are not significantly different from that of EGF.

## Discussion

Our results demonstrate that Th2 cytokines increased in several conjunctival, especially allergic, diseases have a direct effect on conjunctival goblet cell function. However, this effect depends on the time and the presence of other mediators, such as histamine or other cytokines.

IL4, IL5, and IL13 are some of the most studied Th2 cytokines in ocular allergy^25^. We showed that these cytokines increased goblet cell proliferation. This fact would explain why goblet cells have increased numbers in allergic conjunctivitis or vernal keratoconjunctivitis (VKC)^26^. The role we found for IL4 and IL13 in stimulating goblet cell proliferation is consistent with their effect on conjunctival fibroblasts^27^. Moreover, we showed that IL13 also significantly increased goblet cell secretion. Thus, in these allergic *in vitro* inflammatory conditions, there are more goblet cells, and more stimuli present to cause goblet cells to secrete their products, leading to mucous overproduction similar to the findings in allergic eye diseases *in viv*o and in a three-dimensional model of human conjunctiva^28^.

IL13 is the Th2 cytokine that showed the most extensive effects on conjunctival goblet cell function. Of the three cytokines studied only IL13, not IL4 nor IL5 stimulates an increase in [Ca^2+^]_i_, glycoconjugate secretion (including MUC5AC), and cell proliferation. As IL13 shares a common receptor with IL4, we also analyzed the potential interaction between both these cytokines. We observed that IL13 blocked IL4-mediated increased in [Ca^2+^]_i_ and interestingly, when both cytokines were added together the response was lower than that induced by IL13 alone. An explanation for these results may be found in the IL13-Rα2, a decoy receptor. Andrews *et al*. revealed that over expression of IL13-Rα2 attenuated IL4 and IL13 mediated STAT6 phosphorylation^29^. The cytoplasmic region of IL13-Rα2 interacts with IL4 receptor, modulating its signaling. When both IL4 and IL13 are added together, a portion of IL13 binds IL13-Rα2 which blocks IL4 signaling through the common receptor, explaining the lower response observed with the IL4-IL13 combined treatment.

Histamine is present in allergic conditions, and in the conjunctiva plays a role in mucin secretion^8^. The effect of Th2 cytokines IL5 and IL13, but not IL4, on histamine-induced increase in [Ca^2+^]_i_ was the opposite of histamine alone, in that cytokine pretreatment blocked the histamine responses. This could be an attempt by goblet cells to regulate oversecretion. This interesting finding shows an effect similar to that of resolvins (Rv), a family of pro-resolving lipid mediators that block inflammatory processes in a wide range of tissues^30^. In conjunctival goblet cells select Rvs, when bound to their receptors, block histamine-stimulated secretion by preventing its increase in [Ca^2+^]_i_ and the further activation of ERK1/2 that lead to mucin secretion^31^. In a second similar mechanism we found that IL5, but not IL13, counter-regulates the H1 histamine receptor through β-ARK1 to prevent histamine dimaleate-mediated increase in [Ca^2+^]_i_. This mechanism is also used by RvD1 and lipoxin A_4_ to prevent the actions of histamine in conjunctival goblet cells^31,32^. Although Th2 cytokines and Rvs show similar mechanisms of action, these mediators are produced under different circumstances. Rvs are always present, but Th2 cytokines are predominantly produced during the allergic response.

Our experiments are performed in cultured goblet cells obtained from rat conjunctiva. In previous studies, rat conjunctival goblet cells were found to behave in a similar manner as human goblet cells^33–35^. Thus rat conjunctival goblet cells are an excellent model for human goblet cells.

In summary, with this study we are emphasizing the importance on goblet cell function in the context of which cytokines are produced. In addition, the specific time lapse when they are produced during the inflammatory and allergic responses is also paramount. We have shown that different cytokines directly affect goblet cell function causing varying effects. They can block an increase in [Ca^2+^]_i_ and secretion or, in contrast, induce goblet cells to proliferate and secrete their products (mainly mucins). In light of our results, the high levels of IL4, IL5, and IL13 that characterize allergic conjunctivitis could be the reason for the increased number of goblet cells and could have a role in the mucin overproduction found in this pathological condition. The fact that Th2 cytokines block the histamine response when this allergic mediator is present, suggests that these cytokines may be playing an important regulatory role, preventing even more mucous production induced by histamine. This counter regulation, especially in the case of IL13, could help to explain why some patients do not respond as well as expected to anti-histaminergic drugs. The reduction of histamine in the goblet cell microenvironment could lead to an increase in secretion mediated by the Th2 cytokines, whereas before the treatment those cytokines were blocking the histamine response. Further research using anti-histaminergic compounds in *in vitro* and *in vivo* experiments will help to clarify this hypothesis. In conclusion, our research highlights the importance for goblet cell function on the environment in which these cells are immersed and the changes in the environment depending on the individual pathological conditions.

## Methods

### Materials

Rat IL4, IL5 and IL13 were from NCI BRB Preclinical Repository (Frederick, MD). Histamine was purchased from Sigma (St. Louis, MO, USA). Fura-2/AM was from Invitrogen (Carlsbad, CA, USA). The 35 mm glass bottom culture dishes were from MatTek Corporation (Ashland, MA, USA). RPMI 1640 cell culture medium, penicillin/streptomycin, and L-glutamine were purchased from Lonza (Walkerville, IL, USA). FBS was from Atlanta Biologicals (Norcross, GA). Alamar Blue was from Thermo Fisher (Waltham, MA, USA).

Primers for rat cytokine receptors were from SABioscience-Qiagen (Frederick, MD, USA). All reagents for reverse transcription polymerase chain reaction (RT-PCR) were from Biotools B&M Labs S.A. (Madrid, Spain), except Blue Juice™ Gel Loading Buffer 10× (Invitrogen, Inchinnan, UK).

### Animals

Male Sprague-Dawley rats between 4 and 6 weeks old were obtained from Taconic Farms (Germantown, NY, USA). All experiments followed the ARVO Statement for the Use of Animals in Ophthalmic and Vision Research, and were approved by the Schepens Eye Research Institute Animal Care and Use Committee. Rats were anesthetized with CO_2_ for 2 min and decapitated. Then, forniceal and bulbar conjunctiva were removed from both eyes.

### Cell Culture

Conjunctival goblet cells were grown in organ culture as previously described^33^. Briefly, conjunctival tissue was carefully minced into small pieces, and placed in six-well plates. When cell outgrowth was observed, tissue explants were removed. Conjunctival goblet cells were cultured from every sample, although goblet cells were not obtained from each piece. As early as 24 h after establishment of organ culture, cell outgrowth from the explants was observed. Cells were fed with RPMI-1640 medium supplemented with 10% fetal bovine serum (FBS), 2 mM L-glutamine, 100 µg/ml penicillin-streptomycin. Cells were maintained at 37 °C and 5% CO_2_, and medium was changed every other day. After 7–10 days, cells were trypsinized and passaged. Cells in passage 1 were used for all the experiments.

### Immunofluorescence microscopy

First-passage cultured cells were grown on glass cover slips and then fixed in methanol for 10 min. To confirm that the cultured cells were goblet cells, they were stained with antibody against CK7 and the lectin UEA-1 conjugated to FITC (Sigma-Aldrich). Cells were incubated for 1 h with a blocking solution. Thereafter primary antibody anti-CK7 at 1:100 dilution was added. After one hour, the cover slips were washed with PBS, followed by addition of a secondary antibody conjugated with Cy3 (1:200 dilution) and UEA-1 (1:500 dilution) for a one-hour incubation. Cell nuclei were detected with 6-diamidino-2-phenylindole (DAPI) added in the mounting medium. Negative controls included the omission of primary antibodies. Cells were viewed by fluorescence microscopy (Eclipse E80i, Nikon, Tokyo, Japan). Micrographs were taken with a digital camera (Spot, Diagnostic Instruments, Inc, Sterling Heights, MI).

### RNA Isolation and Reverse Transcription PCR

To determine the genetic expression of cytokine receptors total RNA was extracted with TRIzol and isolated according to manufacturer’s instructions (Invitrogen, Carlsbad, CA). Complementary DNA (cDNA) was synthesized from one microgram of purified RNA using the Superscript First-Strand Synthesis system for RT-PCR (Invitrogen, Carlsbad, CA).

RT-PCR was conducted using MyGene™ Thermocycler. Each reaction contained 50 ng of cDNA, 1 µl of primers, 5 µl of PCR buffer 10×, 1 µl dNTPs, and 1 µl Taq polymerase, in a final volume of 50 µl. Non template controls (NTC) included the omission of cDNA. Thermocycling conditions were 95 °C for 2 min, followed by 39 cycles of 95 °C for 20 s, 60 °C (primer-specific annealing temperature) for 30 s, 72 °C for 40 s and a final cycle of 72 °C for 10 min. RT-PCR products and the 100 base pair ladder markers were mixed with 2 µl of Blue Juice™ Gel Loading Buffer 10x and were resolved on 4% agarose gels. Gel images were captured with the ChemiDoc® gel documentation system.

### Measurement of [Ca^2+^]_i_

First-passage cultured goblet cells were grown on 35 mm glass-bottom culture dishes for 1 day. Cells were then incubated in KRB buffer (containing 120 mM NaCl, 25 mM NaHCO_3_, 10 mM HEPES, 4.8 mM KCl, 1.2 mM MgCl_2_, 1.2 mM NaH_2_PO_4_, 1 mM CaCl_2_) with 0.5% BSA, 8 μM pluronic acid F127, 250 μM sulfinpyrazone, and 0.5 μM of Fura-2/AM for 1 h at 37 °C. When activated Fura-2 is a fluorescent molecule that indicates the intracellular [Ca^2+^]. After incubation cells were washed with KRB buffer containing sulfinpyrazone, and the dishes were observed using a Ca^2+^ imagining system, InCyt Im2 (Intracellular Imaging, Cincinnati, OH, USA). This system allows measuring the ratio of Fura-2 using excitation wavelengths of 340 and 380 nm, and an emission wavelength of 505 nm. A mean of 30 cells per dish was selected, and [Ca^2+^]_i_ was measured in each individual cell. A basal reading was done for at least 15 seconds. In select experiments histamine (10^−5^ M) known to produce an increase in [Ca^2+^]_i_ was used as a positive control as well as an example of an allergic inflammatory mediator. The effect of IL4 (10 ng/ml), IL5 (10 ng/ml), and IL13 (10 ng/ml) cytokines on [Ca^2+^]_i_ in cultured goblet cells was evaluated by adding 100 µl of each cytokine. Additionally, the effect of IL4, IL5, and IL13 on histamine-induced increase in [Ca^2+^]_i_ was studied after a 2.5 min, 15 min or 24 h exposure to each cytokine followed by addition of histamine. Data are presented as the change in peak [Ca^2+^]_i_, that was calculated by subtracting the basal value from the [Ca^2+^]_i_ peak.

### High Molecular Weight Glycoconjugate Secretion

For secretion assays, first-passage goblet cells were cultured in 24-well plates and grown to confluence. Before using the cells, they were serum starved for 24 h. Then, they were incubated with buffer alone (basal), histamine (10^−5^ M), or cytokines (10 ng/ml for IL4, IL5, and IL13) for 2 h in serum-free RPMI 1640 supplemented with 0.5% bovine serum albumin. The 2 h incubation time was selected based on our previous studies^8^. Goblet cell secretion was measured using enzyme-linked lectin assay (ELLA), from Pierce Biotechnology (Rockford, IL, USA). UEA-1 lectin conjugated to horseradish peroxidase was used to detect high molecular weight glycoconjugates, including the mucins that are produced by goblet cells. After incubation, the culture medium was collected and the amount of lectin-detectable glycoconjugates was measured. To perform the ELLA, standards and supernatants were placed into 96-well microplates and dried overnight at 60 °C. Manufacturer’s protocol was followed. The UEA-1 was detected using Amplex Red (Invitrogen, Carlsbad, CA). In the presence of hydrogen peroxide, Amplex Red is oxidized producing a fluorescent molecule. That fluorescence was then quantified using a fluorescent ELISA reader (Bio-Tek, Winooski, VT), using 530 nm and 590 nm excitation and emission wavelengths, respectively.

After collection of supernatant for ELLA, cells in the 24-well plate were removed and sonicated. The cell homogenate was analyzed for total amount of protein using the Bradford protein assay. Bovine submaxillary mucin was used for the standard curve.

The amount of high molecular weight glycoconjugate secretion was normalized to total protein in the homogenate, and expressed as fold increase over basal. Basal value was set at 1.

### Proliferation

Proliferation was measured using Alamar Blue assay in six independent experiments. Briefly, cells were serum starved for 24 h, treated with EGF (10^−7^ M), histamine (10^−5^ M), IL4 (10 ng/ml), IL5 (10 ng/ml), or IL13 (10 ng/ml) for 24 h. After stimulation 10% Alamar Blue was added to the wells and after a 4 h incubation, fluorescence was read in a spectrophotometer, following manufacturer’s instructions.

### Data Presentation and Statistical Analysis

Data were presented as mean ± standard error of the mean (SEM). To compare two groups, a Student’s t-test was done. For more than two groups, a one-way analysis of variance (ANOVA) was done after assuring equality of variance (Levene’s test). After that, pairwise comparisons (Tukey test) were performed. In the absence of variance equality, a non-parametric test was performed (Kruskal-Wallis test). Differences were considered to be significant when p ≤ 0.05. The primary data is available as supplementary information.

### Data availability

All data generated or analyzed during this study are included in this published article (and its Supplementary Information files).

## Electronic supplementary material

Supplementary material
