# Supplementary material for: Context-Dependent Regulation of Conjunctival Goblet Cell Function by Allergic Mediators
Source: Sci Rep. 2018 Aug 15;8:12162. doi: 10.1038/s41598-018-30002-x (PMC6093861; doi:10.1038/s41598-018-30002-x)
Supplement: Supplementary file 1 — Supplementary material [file 41598_2018_30002_MOESM1_ESM.pdf]

Context-Dependent Regulation of Conjunctival Goblet Cell Function by Allergic Mediators

Laura García-Posadas, Robin R. Hodges, Yolanda Diebold, Darlene A. Dartt

SUPPLEMENTARY MATERIAL

TABLE S1. Histamine and Cytokines Increase in $[Ca^{2+}]_i$ – Time Traces

Time	IL4	IL5	IL13	Histamine
0,59	339,3697431	263,0182098	256,3190985	280,3288162
1,42	334,9705706	263,7073065	252,9878676	278,7195194
2,25	335,3398845	260,671526	253,0282004	279,2148858
3,07	333,9951821	257,8523979	256,3281941	277,2999481
3,9	328,7322638	259,4068373	256,4173797	277,6352226
4,73	330,7144808	260,9119247	252,2619589	274,4355134
5,55	334,4440237	259,8191938	254,0576464	272,9765171
6,38	330,6897747	259,3426396	252,8755334	275,2578386
7,21	330,8647802	260,7351059	255,0516652	274,5025425
8,03	324,2427361	257,6188559	252,2791176	275,3657613
8,86	329,6287735	260,9356372	252,4146265	272,0541212
9,69	323,2242143	258,0361142	246,8010307	273,6983367
10,51	326,4764671	259,9663283	247,7865922	270,7751803
11,34	326,4776719	258,3554487	247,4747253	268,1217572
12,17	326,18629	258,006825	250,3917829	272,1833919
13	326,0970674	256,6836871	249,901565	268,2954999
13,82	323,0449689	257,2181097	248,807601	257,9641481
14,65	298,2723679	256,0742584	239,4137276	271,1820759
15,48	326,0153517	251,6319439	239,7466192	280,9812866
16,3	339,3589108	254,8574269	247,5839673	278,5358932
17,13	320,2683042	253,773274	251,5299745	308,6871009
17,96	328,0262272	248,174429	264,9284358	322,4591665
18,78	323,5523295	258,086085	268,1534533	329,6793555
19,61	328,4195652	267,4813783	274,8838505	344,8060115
20,44	335,371897	268,5527868	289,0574452	351,8136476
21,26	334,769673	272,1910759	298,9481607	356,9631311
22,09	335,2579126	277,9648297	307,0696532	362,9291716
22,92	339,9018852	278,9514749	316,9278947	374,7013723
23,74	333,2650768	282,2471066	319,3002789	377,9248922
24,57	338,8501423	283,6154687	321,057503	390,7211705
25,4	340,1796919	286,913622	326,9936321	394,7211834
26,22	340,4552956	288,9645749	330,0123859	397,0548787
27,05	340,6927236	290,7686212	332,4932201	406,2930311
27,88	341,6305548	290,9929853	335,5877618	404,9125713

28,7	344,6231508	293,9993648	337,7642594	419,1090422
29,53	344,9185761	292,1900303	345,6378046	418,1176422
30,36	342,5387998	292,6120364	343,3416549	426,0820986
31,19	346,3859916	297,2166655	347,2366666	426,2556315
32,01	343,151785	295,82719	350,7351024	431,5713851
32,84	349,626165	296,3782863	353,2564967	432,9285072
33,67	344,8892015	295,9750945	352,8963202	434,4339147
34,49	345,9261229	299,0005478	355,8231681	435,5234223
35,32	349,0282531	293,7158544	354,2690503	436,4145402
36,15	348,3228736	299,8528736	355,9596376	435,4105961
36,97	347,0165536	294,3835802	356,9592582	438,9347208
37,8	349,4306565	297,3614808	357,0048244	436,1066027
38,63	348,0688576	297,8203924	355,2576532	436,1878412
39,45	347,0566587	299,332704	357,1495213	437,3285057
40,28	348,699525	298,5688869	352,4541865	435,6439181
41,11	346,5337427	298,2586804	358,0749974	434,8718549
41,93	347,4788563	295,0769437	359,0011652	435,2516907
42,76	348,7738784	299,2491914	353,0311089	434,0647413
43,59	348,9179286	295,5417261	352,2669911	431,4527362
44,41	346,811512	297,9648547	355,2081946	433,9097783
45,24	348,6908497	293,6047207	354,4715734	433,8230094
46,07	346,1703734	297,6487273	354,7222388	426,4567076
46,89	350,6200414	293,7782376	353,6756572	424,8581718
47,72	349,607428	296,1245218	350,0120612	426,7382009
48,55	349,6674005	294,4594472	351,4955871	422,6935736
49,37	350,1735149	294,9149078	349,4999037	422,7261265
50,2	345,9974659	296,475056	352,6215881	418,0201131
51,03	347,6168728	295,2251036	348,7094093	423,4391227
51,86	347,9622862	293,182388	353,4817169	421,5946494
52,68	348,2562382	293,0897907	349,3948758	425,9082958
53,51	347,4999834	294,9119163	351,3123388	425,1222917
54,34	347,036924	294,2734091	352,3375528	421,5937111
55,16	345,7982146	292,8141763	349,1498152	421,2725725
55,99	347,8287568	294,5601784	343,9035627	416,208037
56,82	347,9046996	293,113969	348,2358633	414,6758054
57,64	344,2107556	292,272412	348,274383	408,4303985
58,47	350,6195442	294,8351876	349,0831425	409,993013
59,3	349,393255	293,5434931	347,9208065	406,3958899
60,12	348,9510622	292,0065553	343,856093	408,5612069
60,95	345,7912301	293,597667	344,0683397	407,980131
61,78	346,4075301	291,2110242	344,4047946	408,0088072
62,6	344,6869851	292,4831871	342,3968806	402,0409179
63,43	345,203678	294,5740503	342,1892374	402,3417641

64,26	346,2145086	292,0885323	344,530094	402,6589371
65,08	344,339311	291,7487667	342,7578199	398,9765748
65,91	345,6719106	292,3631063	344,551793	401,3383178
66,74	346,0409246	290,9619746	344,6620374	396,3050508
67,56	345,4945474	287,6567691	344,9587653	401,2949578
68,39	345,7167975	291,4726853	344,9832941	402,4168971
69,22	344,046771	291,288515	345,2010651	398,5508505
70,04	345,607171	292,8340908	343,1767707	398,7871655
70,87	342,5974267	290,7857513	341,0153552	399,9446055
71,7	344,7497121	290,7613561	339,486083	396,9330282
72,53	343,2367146	292,8098114	339,7335102	396,6854096
73,35	346,983148	289,6620333	340,8509714	390,2511735
74,18	346,2791678	290,8320788	340,0792422	393,6126564
75,01	343,8252378	291,5601215	341,7909399	394,3243895
75,83	342,9376089	292,2366926	335,8997666	393,9923132
76,66	343,1574604	288,7410783	335,6517195	389,8550346
77,49	344,847511	292,0468521	341,058204	389,927871
78,31	342,7289133	289,2970306	341,0549712	387,9911331
79,14	340,8043841	287,6441568	335,7129876	386,5022535
79,97	343,4679419	289,817704	339,3346966	388,8754562
80,79	338,8532977	286,5220551	338,753354	386,4794775
81,62	341,3378152	288,7388832	335,9501292	387,0495788
82,45	343,146945	287,3211044	336,053577	387,5335097
83,27	341,5535454	288,5649696	338,2059687	382,3648098
84,1	342,1470166	289,0663452	337,7091064	384,6476512
84,93	340,9308156	288,3989071	340,3271259	383,5983001
85,75	341,7958579	288,5347532	337,2040896	382,6753306
86,58	346,650087	287,5337102	336,4262159	381,4774399
87,41	340,9737898	287,4231182	341,0075117	378,9202513
88,23	345,0067549	290,731924	335,8982117	379,2231634
89,06	347,3224862	288,2413837	338,0118883	379,5652219
89,89	338,152713	289,5903742	337,40036	377,4876567
90,71	340,7147415	289,8060939	332,8328284	377,051253
91,54	342,7750706	288,7767147	338,1942633	376,5782094
92,37	345,3114647	290,7449092	337,8302707	378,0887279
93,2	342,9347524	286,1805766	334,6195615	376,8736333
94,02	339,2959346	287,8624598	337,5771282	374,593395
94,85	345,0424868	290,2617401	339,6627863	374,5619692
95,68	343,0265634	287,5799559	335,405399	371,820947
96,5	342,7918657	287,6037313	334,6647947	372,1655766
97,33	342,5833725	289,3417282	337,6159222	375,3428848
98,16	344,3934034	286,9719578	334,2004283	370,8705041
98,98	340,5246506	287,8457129	332,1131369	371,5938573

99,81	340,8489996	287,0768756	336,1248455	367,8312874
100,64	342,9166541	288,2500604	334,4268471	372,7132482
101,46	342,0492056	288,5257828	336,0152199	369,1993292
102,29	340,6012221	289,7086401	334,0620536	368,5565076
103,12	342,9581187	288,2359632	332,6268251	370,1771463
103,94	343,1428984	287,4832316	336,4075738	
104,77	343,4795974	285,7055093	332,4732459	
105,6	340,0519975	289,9485055	333,9560748	
106,42	342,8846887	290,3245233	336,9568106	
107,25	339,5771001	290,9266146	333,4545993	
108,08	342,1178832	287,9662971	329,0302759	
108,9	343,4133225	291,0711572	335,0429264	
109,73	343,1431615	289,720774	332,5145401	
110,56	343,9861727	288,0412587	333,9476196	
111,38	342,9042391	289,781741	330,3640831	
112,21	343,5019959	288,5901115	331,5854311	
113,04	342,632719	288,3074227	329,6495104	
113,87	339,810039	288,9113296	328,3903786	
114,69	341,8849773	289,04288	334,9543474	
115,52	343,708432	286,14127	332,2584667	
116,35	342,1224714	286,0789259	331,4937391	
117,17	338,1370447	289,9113956	335,359852	
118	341,8581266	289,2065193	329,9035717	
118,83	339,0338678	285,4033453	330,7787126	
119,65	342,8229056	289,1002778	329,8023437	
120,48	338,8072938	290,3507583	333,4097482	
121,31	340,1572735	286,8252145	330,3813044	
122,13	340,4900267	286,4768761	330,6535134	
122,96	342,2294191	288,564355	327,7668412	
123,79	338,3759714	286,7814079	333,2712505	
124,61	339,9702166	287,1259587	331,8830887	
125,44	338,0839473	288,7573693	328,4945151	
126,27	342,4762088	285,6436374	331,2645458	
127,09	339,8068993	287,0751083	326,9025041	
127,92	338,7697099	283,1728322	329,5499154	
128,75	340,6040258	290,0311008	330,4825609	
129,57	339,12873	286,2987105	332,4647356	
130,4	335,2176468	285,8248858	330,5132976	
131,23	336,6572357	286,9352783	330,7310866	
132,06	335,3079066	288,0591211	329,1853578	
132,88	336,7724076	287,2130628	328,9118361	
133,71	341,2023914	285,5215086	328,9063846	
134,54	336,7919271	287,8373191	331,2578311	

135,36	338,9669171	285,8926118	328,6185826
136,19	335,3836048	288,9016979	330,0120858
137,02	342,4528086	289,3759874	328,6368879
137,84	338,8201373	288,0660604	327,1809029
138,67	332,3356153	286,8454377	329,5996857
139,5	334,8983448	287,2520123	325,8228301
140,32	335,391146	285,9877592	329,1386928
141,15	340,6424853	285,4123278	329,4721685
141,98	337,0237201	289,6021492	327,2101484
142,8	334,3760711	287,3373187	329,3486344
143,63	336,4419937	289,4537383	329,9394462
144,46	339,2922836	287,2369209	329,5451799
145,28	340,4855465	289,1244958	325,960438
146,11	335,4435545	287,5280094	328,8478009
146,94	338,7753322	287,918543	330,3082164
147,76	333,9097242	285,8583186	329,7375949
148,59	337,8256778	289,0534291	325,629279
149,42	342,7642379	286,8714407	327,5724448
150,24	336,4123447	289,354741	329,1214144

TABLE S2. Histamine and Cytokines Increase in $[Ca^{2+}]_i$ – Peak $[Ca^{2+}]_i$

Basal	IL4	IL5	IL13	Histamine
0	98,80769231	74,09090909	71,86956522	264,24
0	98,1	114,65	108,29	213,3
0	123,38	215,575	358,07	479,8
0	148,3571429	51,76923077	466,4642857	279,3717949

TABLE S3. Effect of 2.5 min IL Incubation on Histamine Stimulated Increase in $[Ca^{2+}]_i$ – Time Traces

Time	Histamine	IL4 + Histamine	IL5 + Histamine	IL13 + Histamine
0,59	253,3924692	343,2132131	232,0578512	253,5863153
1,42	250,6563922	338,0847608	230,7187896	250,8800533
2,25	251,952418	338,6698459	228,6989394	250,5094396
3,07	248,5612801	334,8142428	227,5872448	252,7589944
3,9	249,4069757	329,9119072	227,5210212	255,5385752
4,73	247,267687	333,9808633	228,1985187	249,624198
5,55	244,5839826	337,2686983	228,1029726	251,048471
6,38	247,7930278	334,3112552	227,757329	249,9180445
7,21	248,3262655	334,5928181	229,0465698	252,852634

8,03	249,2724253	326,3896481	225,7529983	249,8939729
8,86	244,1389626	332,8859202	229,3222781	248,8950882
9,69	246,278753	326,7058413	226,7221999	244,1514203
10,51	243,9132868	332,4239561	227,8001045	244,700123
11,34	239,5805236	332,5848959	226,4420268	243,6288751
12,17	244,7152124	330,7334977	226,076481	246,8984922
13	240,2004712	331,3296454	225,0308685	245,7285234
13,82	227,2480094	327,6195141	223,9855748	243,8459048
14,65	246,1026884	316,8598239	220,083535	231,357269
15,48	257,7250855	336,16558	220,4740205	232,5767336
16,3	269,8037184	349,671881	224,7789501	242,3600023
17,13	288,4265803	324,2659612	221,5279367	251,2536297
17,96	303,6135162	332,4136363	215,4240005	248,6429259
18,78	315,197419	325,3635505	229,1438276	250,169524
19,61	326,9316234	333,0125314	241,127314	245,2741685
20,44	334,1029832	340,2416405	244,1313348	256,5467546
21,26	337,5791809	339,2648973	245,9390536	263,5701453
22,09	345,3116855	339,2307723	254,4271539	267,5111928
22,92	358,37487	343,0709581	257,6752998	277,3299745
23,74	360,0389162	337,1592135	260,5975707	275,5484638
24,57	376,5804801	342,476412	261,5927677	279,9072684
25,4	379,2575486	344,4084781	264,5657817	281,3471417
26,22	380,3450519	344,5355052	268,4237189	282,7550433
27,05	392,1442796	343,6780759	269,8331616	284,8016497
27,88	389,7743686	345,6656286	270,4696946	290,4814754
28,7	408,0519219	349,5264234	272,6729625	287,826139
29,53	405,5694998	348,168546	273,1693261	294,5239464
30,36	414,6307835	346,6659553	273,0455724	293,6725054
31,19	413,5590716	350,5106555	277,260792	296,3380727
32,01	421,3671398	345,2910467	277,9360152	300,7659526
32,84	421,2547861	351,5495533	278,6986674	302,1976738
33,67	421,4892733	348,8598242	277,2844117	300,6876339
34,49	422,4744714	350,3794972	279,5366828	302,4163391
35,32	421,3708155	353,2972264	274,875425	302,6669176
36,15	420,025514	352,4051647	283,6335934	305,1954938
36,97	424,4212578	348,3338493	275,2740594	304,0422522
37,8	420,2139318	351,8453198	279,5581649	304,1786165
38,63	418,1623047	350,6333657	279,8645708	306,5016756
39,45	419,7531175	348,6655449	281,837653	307,3541893
40,28	418,4654928	352,3042556	278,1089921	303,2762487
41,11	415,7706355	349,0869903	279,9137167	305,1773069
41,93	416,3561675	350,4400307	276,3899726	306,2939444
42,76	416,8730861	351,6816157	280,6236838	305,2336165

43,59	411,6388925	350,2996826	277,2796824	302,3938501
44,41	414,3591123	347,4062383	279,9195681	305,1178917
45,24	416,9161981	350,6491329	274,5386753	306,3550174
46,07	405,9516113	349,025609	279,4823507	301,221169
46,89	405,0030997	355,295833	274,3259835	302,0362096
47,72	409,1319114	352,2605707	275,6726957	299,3408172
48,55	404,245186	351,6776451	275,504263	300,1783691
49,37	404,8432541	352,0009088	274,139639	298,9520785
50,2	398,0014207	347,7477323	275,2476937	303,0244163
51,03	405,6095568	349,5107193	276,2994239	296,5378102
51,86	403,545955	348,5974928	273,0562792	303,8018409
52,68	411,1951637	350,4598732	272,0321019	299,6631448
53,51	410,4249421	349,433089	274,7989836	298,4942219
54,34	405,9840751	346,5307875	274,7614502	298,1620704
55,16	405,2257743	346,2829529	271,4389018	298,2762594
55,99	398,9810029	349,8163424	274,2233331	295,5281295
56,82	397,9783266	348,7215995	275,0610063	298,8169211
57,64	389,605049	345,4223408	271,5860731	296,809798
58,47	392,7770637	350,3045034	272,5071548	296,1683509
59,3	389,3673526	349,4510067	271,146086	297,9436041
60,12	392,3005591	348,5098608	271,1963595	294,6758481
60,95	391,9226227	347,3227512	271,6314132	292,8631886
61,78	390,3039651	346,8371512	268,619937	294,5230364
62,6	383,9075486	345,7470913	272,9156781	291,0746914
63,43	385,9368882	345,0620151	273,969448	292,4263166
64,26	386,8720016	347,7246781	271,1118526	294,3357116
65,08	380,7592182	344,7395258	271,2821651	293,485461
65,91	384,5101685	345,2312142	273,4386656	294,3403907
66,74	380,6453059	346,1936773	269,2173947	293,937705
67,56	386,2774774	347,3580631	265,7671208	292,567963
68,39	388,236162	344,9026189	269,1685804	291,6394956
69,22	383,339681	344,213028	269,6413533	292,4479948
70,04	384,0336077	345,0753391	272,7064068	291,480108
70,87	384,4791871	344,5994579	268,6936208	292,6531403
71,7	382,3776762	345,4062827	267,7127605	290,3448923
72,53	381,2113031	343,9929529	270,5673676	289,9484964
73,35	375,4829138	347,1919751	267,6362825	289,9289964
74,18	379,3531755	346,390446	270,2242003	287,9606908
75,01	380,4085511	343,0780948	269,627781	289,0913681
75,83	378,966558	344,0317008	268,6767806	285,9097808
76,66	374,2804734	344,3730583	266,0438187	284,4040858
77,49	375,7133701	343,9415702	270,4793742	288,9239731
78,31	372,7644718	342,8538844	266,0791361	291,2335938

79,14	370,3134931	341,0249566	263,9983996	286,3325352
79,97	372,489369	342,6245892	267,7814624	287,3830668
80,79	371,887246	337,6979525	262,5820259	285,1080812
81,62	370,7377412	339,3255314	265,4942252	284,2655975
82,45	373,0759066	342,2303712	263,4174249	286,1986774
83,27	366,4605828	343,5320606	265,2468642	287,4698204
84,1	369,9506533	342,4382444	264,5622698	283,8622568
84,93	367,0423672	340,8648652	265,2478285	287,3492484
85,75	366,4036459	342,8000327	263,8380042	284,5255678
86,58	364,9651946	346,6316715	264,9358993	285,9973453
87,41	362,3407197	339,867053	262,7924909	284,1066363
88,23	361,9042728	348,4261177	267,0894701	285,2598915
89,06	362,9806255	347,6064261	264,9156545	284,3227016
89,89	359,0322785	337,6727285	265,6945466	284,0415605
90,71	360,1515913	339,4260997	264,010268	281,9608516
91,54	358,921361	342,2658719	264,4275244	281,172397
92,37	361,7394211	344,7403974	266,8177361	287,4776253
93,2	360,0059127	342,3532255	262,331483	282,0757946
94,02	357,3424387	338,7496905	264,6018512	282,7158491
94,85	355,4781995	346,6822046	264,8525582	283,199922
95,68	354,2814642	343,4649735	264,7335126	280,772578
96,5	354,3437846	343,8002654	263,412118	280,924324
97,33	358,4739746	344,3704966	263,8049234	282,5658043
98,16	352,9405989	342,965649	261,7683247	278,6609848
98,98	353,7552164	340,5444231	262,7947601	279,095309
99,81	348,5315028	342,0028884	261,2736913	280,8428514
100,64	355,6323652	342,1190943	260,9372233	282,9383709
101,46	349,8137698	342,2331631	263,6424723	279,2069139
102,29	350,3950199	339,5507406	265,3189011	276,9047382
103,12	353,1480632	343,8526027	263,2165224	277,8483186
103,94		342,4363089	261,6219278	278,8699145
104,77		342,5556855	260,5413934	276,4296382
105,6		338,4115522	265,4884835	280,3570652
106,42		341,4591405	265,1846025	278,6317475
107,25		338,3639113	266,1581052	279,8113049
108,08		341,377622	262,1674437	275,2303449
108,9		341,6866522	263,7877335	276,779534
109,73		342,9746598	262,0865082	276,8333868
110,56		341,1246747	260,0892974	276,8841365
111,38		341,2860966	263,1285118	273,4579499
112,21		343,5091057	263,4722915	276,2792645
113,04		342,1538475	262,4327541	272,2943357
113,87		337,9942742	262,4803443	272,3762175

114,69	339,7646364	261,6359829	275,2684861
115,52	342,488576	259,0331219	276,5415419
116,35	343,1112951	258,0257108	275,4124567
117,17	335,8678374	263,2349561	278,4570671
118	341,7219465	263,6563115	272,3185554
118,83	337,8713792	259,0180319	274,8736628
119,65	341,5049852	260,0944181	273,6225502
120,48	337,6879473	264,6229159	273,1080321
121,31	339,3583647	260,6405241	273,6652565
122,13	339,8949245	259,6327396	273,6421328
122,96	341,3734478	261,9915209	269,2389607
123,79	335,7504063	260,40402	272,2009086
124,61	339,7022887	260,1058021	272,870854
125,44	337,1688186	261,5469685	270,8053764
126,27	340,0956117	257,0422308	271,7778081
127,09	339,4294214	258,4075254	268,5193617
127,92	336,8280576	256,2323477	270,5076114
128,75	338,6998122	262,2617058	268,9623801
129,57	337,4034178	257,2418521	272,7548428
130,4	334,0590846	258,2569906	270,6273853
131,23	333,160092	260,2653711	271,9152189
132,06	331,7427643	260,6252568	270,6463391
132,88	333,2992101	258,6440837	270,8411608
133,71	339,9874107	257,8451067	269,1382369
134,54	333,9107916	258,9371397	270,533407
135,36	335,6812228	258,3482442	270,5451906
136,19	331,6405842	261,3167877	270,9638615
137,02	340,247967	261,9296499	268,5748965
137,84	334,297072	260,59189	268,2302154
138,67	329,9437093	258,7303455	270,043512
139,5	329,5869041	258,5145878	267,0338884
140,32	332,1810835	258,1665361	269,3195444
141,15	336,4755359	256,4509609	270,6328913
141,98	334,4320713	262,6842942	266,5223588
142,8	331,9058726	258,8830916	270,7910527
143,63	334,2419917	261,3518891	269,3222271
144,46	336,6906004	259,9716089	270,4629526
145,28	336,9745065	262,3259944	265,9075725
146,11	332,0556283	258,2013935	269,6077115
146,94	334,9017763	258,9642478	266,5811161
147,76	328,3738544	256,1263296	265,7870921
148,59	332,3989037	261,3190959	263,3033375
149,42	338,5207616	256,9852542	264,2606391

150,24	330,7444596	259,6577499	271,599633
151,07	334,39444	257,5867104	262,5903987
151,9	330,8044148	258,878408	268,13516
152,73	330,1896788	253,1839958	304,7045283
153,55	329,9062431	256,2388671	266,4405302
154,38	333,2341253	258,4691244	266,2012403
155,21	326,9359388	258,7027214	267,1223598
156,03	327,4901099	256,9857647	265,9975693
156,86	328,8864716	257,4866509	271,4760397
157,69	329,2929997	260,2970175	269,7460588
158,51	334,0382034	256,1263866	267,6199599
159,34	327,9283961	260,1777649	268,0966355
160,17	329,7404632	253,9028023	268,093537
160,99	330,346188	261,8497196	268,0957624
161,82	331,3627247	258,3910033	267,9499037
162,65	329,2985474	260,5303907	270,2080306
163,47	335,4025872	256,5060535	264,7995573
164,3	330,1606803	259,3694356	265,2324359
165,13	292,028541	260,7069853	267,1072764
165,95	331,2781896	251,9583601	262,4613334
166,78	319,8857106	257,538373	274,3728083
167,61	328,1050321	259,2087672	272,7385237
168,43	346,0014623	259,0554499	268,403051
169,26	349,8185918	261,6383563	273,9221984
170,09	351,0002099	262,8011976	277,6221697
170,91	356,5566898	264,7049021	286,2204061
171,74	358,1199938	265,0601709	283,3852526
172,57	357,9163761	268,9996495	287,1098985
173,4	367,297987	268,679848	286,6989763
174,22	364,6484774	269,4904377	290,1918388
175,05	369,6429229	269,9241163	285,4741202
175,88	379,2598177	271,9686501	287,565647
176,7	384,4507065	274,6020013	286,1846333
177,53	384,7376113	270,9170159	288,067844
178,36	390,3967587	272,0227441	285,7535402
179,18	392,551564	272,6032228	287,8345669
180,01	390,7393166	276,3756893	289,7441799
180,84	393,5008943	278,5889183	284,897063
181,66	395,7163517	273,9173866	286,2589905
182,49	386,9033116	273,6259105	286,8497294
183,32	394,5559457	278,2757312	286,7131133
184,14	394,9037476	273,7548154	287,5652915
184,97	393,609249	278,9874905	284,1471066

185,8	396,1321271	277,3711528	286,5228529
186,62	397,9914744	278,5887902	282,7387423
187,45	394,4260219	278,2600707	284,5501266
188,28	401,3257954	278,885069	279,1813916
189,1	400,00376	277,7780209	284,8300674
189,93	399,3662783	276,6704157	282,6401099
190,76	393,0233926	281,9485649	283,2591298
191,58	394,1633781	281,4179064	283,4468723
192,41	396,595651	284,050021	283,569247
193,24	397,4470375	280,5961924	279,1809031
194,07	396,2166067	283,737779	283,1722219
194,89	398,3079642	282,8097111	278,4653561
195,72	391,786901	281,7296068	282,9382072
196,55	397,0593307	287,3183032	283,0301701
197,37	391,812013	282,7089971	278,6532661
198,2	393,9369727	286,9804927	281,3649121
199,03	397,7945623	283,9747951	280,8030596
199,85	392,5866022	283,2516914	278,9950441
200,68	394,6529735	285,9899514	279,0682709
201,51	391,1043431	280,2449449	278,2329965
202,33	396,3298639	285,1773183	278,8756708
203,16	388,7679956	286,5639718	280,8450832
203,99	390,8795489	290,6984716	
204,81	389,0414189	287,6193088	
205,64	390,9993672	285,6586179	
206,47	389,295336	287,513248	
207,29	391,6951093	282,7501425	
208,12	382,665051	286,7778127	
208,95	384,2679884	287,0026155	
209,77	388,9279628	285,412322	
210,6	380,7127309	285,8526934	
211,43	389,224615	284,5054336	
212,25	384,6777381	282,0081867	
213,08	383,5318473	284,9946513	
213,91	381,1024576	286,4336802	
214,74	384,8207103	285,4718194	
215,56	387,5689123	283,4897144	
216,39	382,1612004	285,9241947	
217,22	383,8448515	283,8421269	
218,04	375,0618638	283,7580132	
218,87	381,1409522	290,3574722	

TABLE S4. Effect of 2.5 min IL Incubation on Histamine Stimulated Increase in $[Ca^{2+}]_i$ – Peak $[Ca^{2+}]_i$

Alone	After IL4	After IL5	After IL13
264,24	79,65384615	49,72727273	119,2608696
213,3	171,5	86,28	20,645
479,8	353,17	183,52	125,43
273,6709402			

TABLE S5. Effect of 15 min IL Incubation on Histamine Stimulated Increase in intracellular $[Ca^{2+}]_i$ – Time Traces

Time	Histamine	IL4 + Histamine	IL5 + Histamine	IL13 + Histamine
0,75	18,6667	7,6	0	0
1,83	18,6667	6,2	0	0
2,91	21,6667	7	0	0
3,98	19,5	7,1	0	0
5,06	18,6667	6,7	0	0
6,14	19,1667	7,6	0	0
7,22	19,5	7	0	0
8,3	18	7,5	0	0
9,38	17,1667	6,6	0	0
10,45	18,8333	6,6	0	0
11,53	17,8333	6,5	0	0
12,61	18	6,3	0	0
13,69	17,8333	6,4	0	0
14,77	18,1667	6,5	0	0
15,84	18,1667	7,1	0	0
16,92	17,6667	7	0	0
18	17,3333	7,1	0	0
19,08	19,1667	6,2	0	0
20,16	18,5	7	0	0
21,23	19,3333	29,6	0	0
22,31	20,3333	46,3	0	0
23,39	19,5	55,8	19,3333	0
24,47	18,8333	68,7	0	0
25,55	0	78,4	2,3333	0
26,63	9,5	87,1	11	0
27,7	19,3333	97,9	17,6667	0
28,78	27	102,5	19,8333	0
29,86	30,5	111,4	23,3333	0,6667
30,94	38,3333	117,2	25,5	0,5
32,02	55,1667	126,9	28,5	1

33,09	67,5	129,9	30,3333	1,5
34,17	82,3333	138,6	34,6667	2,1667
35,25	104,5	144,2	37,1667	2,5
36,33	106,8333	150,6	36,8333	3
37,41	137,1667	154,4	41	4
38,48	141	160,5	46	4,3333
39,56	194,5	160	43,8333	4,6667
40,64	213,5	170	47,1667	2,6667
41,72	222,8333	173,4	45,3333	5,8333
42,8	230	173,7	51,8333	7
43,88	233,3333	180,5	56	7,5
44,95	239,5	182,5	51	7,8333
46,03	242	183,9	49	8,5
47,11	246,3333	191,8	47,8333	9,5
48,19	243	184,3	48,1667	10,3333
49,27	239,3333	186,4	48,3333	11,1667
50,34	237,5	191,9	46,6667	12,3333
51,42	246,1667	195,9	48,3333	12,3333
52,5	246,8333	196,8	49	13
53,58	246	194,7	45,6667	13,3333
54,66	255,3333	197,6	46,1667	14,1667
55,73	264	194,1	45,8333	13,8333
56,81	262,5	195,4	44,1667	14,6667
57,89	256,6667	196	46,6667	16,6667
58,97	253,6667	194,2	46,6667	15,6667
60,05	260,8333	193,4	46,5	16,8333
61,13	265,3333	193,6	44,8333	17,6667
62,2	265	193,7	45,3333	18,3333
63,28	263,1667	189,9	44,8333	18,3333
64,36	258,5	187,7	43,5	19,1667
65,44	264,8333	184,5	41,5	19,6667
66,52	256,3333	184,7	44,5	19,8333
67,59	260,1667	181,5	42,3333	20,6667
68,67	250,8333	185,9	41,8333	20,8333
69,75	248	188,8	43,6667	20,8333
70,83	243,5	181,2	43,3333	21,1667
71,91	244,3333	185,5	40,8333	21,5
72,98	245,5	186,4	38,8333	23
74,06	245,3333	189,5	39,3333	22,3333
75,14	248,3333	188,1	39,8333	18,5
76,22	244	189,4	36,8333	20,1667
77,3	243	194,6	37,6667	20,8333
78,38	244,3333	191,7	36,5	20,6667

79,45	244	187,9	35,8333	21,5
80,53	244,3333	190,1	35,5	22
81,61	242,1667	193,2	36,6667	21,8333
82,69	242,3333	193	33,6667	22,5
83,77	240,6667	185,6	34,6667	22
84,84	239,6667	188,3	34	23,3333
85,91	241,5	185,5	32,8333	22,8333
86,98	236,6667	187	34	23,3333
88,06	245,5	185,5	33	23,6667
89,14	237	183,8	31	23
90,2	233	183,4	32	24,3333
91,28	233,8333	183,6	32,1667	23,5
92,34	233,5	181	31,6667	23,6667
93,41	231	181,5	31,5	24,1667
94,47	232,1667	178,6	30,8333	24,6667
95,55	236,1667	179,4	31,5	23,5
96,61	226,8333	180,1	30,1667	22,8333
97,69	231,5	172,4	27,3333	23,5
98,75	228,6667	179,8	30,6667	23,6667
99,81	227,8333	179,8	30,6667	23
100,88	230,1667	178,3	28	22,3333
101,94	221,6667	180,7	26,6667	21,8333
103	218,5	177,8	28	22,1667
104,06	215,5	177,7	29	22,5
105,13	214,6667	173,2	30,1667	22,1667
106,19	219,1667	176,7	26,6667	22
107,25	222,8333	174,5	27,6667	22,6667
108,31	223	176,1	26,8333	22,8333
109,38	224,1667	174,1	28,6667	22,1667
110,44	218,5	173,3	27,5	22,8333
111,5	221,8333	174,4	24,6667	22,6667
112,56	218,1667	174,9	26,1667	21,6667
113,63	214,3333	173,3	26,6667	21,5
114,69	213,8333	177,6	27,5	22,1667
115,75	212,1667	171,7	25,1667	22
116,81	213	172,1	24,3333	20,8333
117,88	208,8333	171,9	25,1667	21,6667
118,94	209,6667	171,7	24	21,5
120	204,8333	172,5	23,1667	21,3333
121,06	199,3333	167,9	23,5	20,5
122,13	199,8333	172,2	22	20,5
123,19	191,3333	177,8	24	20
124,25	193,6667	167,5	21,8333	20

125,31	194,5	167,8	21,5	20,8333
126,38	189,5	166,9	21,3333	20,3333
127,44	191,5	169,3	20,8333	19,8333
128,5	185,8333	169	21	19,5
129,56	185,5	160,6	20,1667	20,5
130,63	181,5	162,3	21,8333	19,8333
131,69	178,8333	156,1	21,8333	19,1667
132,75	179,8333	156,1	19,3333	19,1667
133,81	178,3333	158,1	20,3333	19
134,88	179,1667	154,7	19,5	18,6667
135,94	175	158,1	20,5	19,6667
137	184,3333	157,2	19,3333	19,1667
138,06	179,5	158,9	18,1667	19,1667
139,13	178	157,7	18,1667	17,8333
140,19	179,3333	159,1	19,5	19
141,25	178,8333	158,5	18,6667	18,8333
142,31	175,5	158,3	17,6667	18,6667
143,38	174,6667	158,2	18,1667	19
144,44	171,1667	158,7	18	18,5
145,5	172,8333	158,7	17,6667	17,5
146,56	175,3333	158	18	18,3333
147,63	174,3333	160,2	16	19,3333
148,69	171,3333	156,2	17,3333	18
149,75	168	154,8	14,3333	17,8333
150,81	165,8333	153,7	16,5	18,8333
151,88	164,6667	163	15,8333	18,1667
152,94	161,8333	156,8	16,3333	18,5
154	169	153,5	16	18,8333
155,06	165,5	155,5	15,1667	18,3333
156,13	165,1667	154	13,8333	18,3333
157,19	159	157,9	14,8333	18,8333
158,25	159	156,4	13,8333	18,1667
159,31	159,3333	155,7	12,6667	18,5
160,38	161,5	157,2	13,6667	18
161,44	158	153,1	12,8333	18,1667
162,5	162,1667	151,6	14	18,3333
163,56	160,5	149,7	14,1667	18,1667
164,63	156,1667	153	13,3333	17,8333
165,69	163,5	154,7	12,6667	18,5
166,75	161,8333	147,3	14,5	17,3333
167,81	164,8333	150,8	13,6667	18,1667
168,88	162,3333	149,5	15,1667	17,8333
169,94	162,6667	147,1	14,8333	17,8333

171	160	147,2	14,6667	18,6667
172,06	158	147,6	14,5	17
173,13	156,8333	145,6	12,6667	17,1667
174,19	158,8333	141	13,8333	18,1667
175,25	156,5	147,4	14	17,3333
176,31	151,6667	142,1	14,3333	17
177,38	149,6667	148,1	14,1667	15,8333
178,44	152,1667	141,3	14,5	15,5
179,5	154,1667	146,3	15	15,8333
180,56	152,5	144,6	13	16,5
181,63	153	145	15,5	16,1667
182,69	152,6667	139,9	14,6667	17,1667
183,75	154	142,7	13,6667	16,5
184,81	152,6667	146,1	13,1667	17
185,88	150,3333	143,7	11,5	17,1667
186,94	146,5	141	15	17,5
188	148	144,9	12,3333	17,1667
189,06	148,5	145,2	12,3333	16,6667
190,13	148,3333	136,1	12,8333	17
191,19	145	144,3	11,3333	17,1667
192,25	141,6667	140	12,6667	17,3333
193,31	148,6667	143,7	12,5	17,3333
194,38	149,3333	142,2	12,8333	17,3333
195,44	148,3333	141,7	9,3333	17
196,5	146,8333	142,2	11,3333	16,8333
197,56	145,6667	141,7	12,5	17,3333
198,63	142	145,2	12,3333	17,1667
199,69	137	139,8	12	18,3333
200,75	143,6667	139,4	10,8333	17,1667
201,81	137,8333	139,4	11,1667	17,5
202,88	141,5	141,8	10,8333	17,8333
203,94	146,3333	143,4	10,8333	16,8333
205	145,3333	141,6	12	17
206,06	145,6667	144,1	10,6667	16,6667
207,13	145,1667	144	12,3333	16,1667
208,19	138,8333	142,9	11	16,6667
209,25	136,3333	140,5	10,5	17,3333
210,31	138,5	140,3	10,6667	16,8333
211,38	138	140	11,3333	16,1667
212,44	134,8333	131,9	10,1667	16
213,5	140,6667	134,3	9,1667	16,3333
214,56	143,5	137,4	10,1667	17,5
215,63	141,3333	138,4	10,5	16

216,69	142,5	136,3	10,5	15,3333
217,75	141,8333	138,7	10,8333	15,6667
218,81	140,5	137,2	11,5	16
219,88	136,5	137,5	11,6667	15,8333
220,94	137,5	132,4	10,5	16
222	141,6667	135,4	9,8333	15,8333
223,06	138,1667	137,4	10,1667	16,1667
224,13	140,3333	130,7	11	16,3333
225,19	137,3333	130,2	9,3333	15,6667
226,25	136	129,8	8,6667	16,1667
227,31	135,6667	129,9	9,3333	16
228,38	137,6667	129	9,1667	15,1667
229,44	134,6667	129,9	10,1667	15,8333
230,5	138,3333	138,4	9,1667	14,8333
231,56	135	129,7	9,1667	15,8333
232,63	137,6667	126,9	8,6667	15,6667
233,69	138,1667	122,5	8,6667	15,6667
234,75	137,1667	125,1	9,6667	15,5
235,81	138,1667	125,3	9,3333	15,6667
236,88	135,5	122,9	8,1667	15
237,94	129,6667	122,2	11,6667	14,8333
239	133	124,5	8,3333	15,6667
240,06	132,8333	124,4	9,8333	15,3333
241,13	136,1667	126,4	8,5	15,8333
242,19	134,6667	131,7	9,6667	15,3333
243,25	130,6667	127,9	9,1667	15,5
244,31	127	125,8	9,1667	15,6667
245,38	125,3333	126	9,3333	15
246,44	126,3333	122,2	8,3333	15,3333
247,5	127,1667	124,6	8,6667	15,6667
248,56	126,3333	122,5	9,8333	15
249,63	124,8333	122,2	9	15,6667
250,69	126,8333	127,8	9,6667	14,5
251,75	122,6667	127,1	9,5	16,1667
252,81	120,3333	125,7	9,6667	15
253,88	116,1667	126,8	7,1667	15,5
254,94	126,5	123,7	8	14,6667
256	132,1667	122,9	10,6667	15,6667
257,06	129,6667	127,1	9,3333	15,5
258,13	128,6667	132,9	10,5	15
259,19	130,3333	128,2	8,8333	15
260,25	131	134,3	8,8333	16,1667
261,31	132,3333	125,2	9	16,3333

262,38	134	126,7	9,1667	15,6667
263,44	134,6667	122,6	8	15,3333
264,5	133,5	128,1	7,1667	15,5
265,56	131,6667	125,8	7,5	15,3333
266,63	130,8333	130,5	9,8333	15
267,69	133,1667	126,3	7,1667	15,1667
268,75	130	126,9	7,8333	15,8333
269,81	133,6667	124,4	10,1667	15,5
270,88	132	125	8,1667	15,6667
271,94	130,3333	128,6	8,8333	16
273	133	128,6	8,8333	15,1667
274,06	130,5	123,6	9,6667	15,5
275,13	123,3333	125,4	8,5	15,3333
276,19	119,8333	124	8,6667	15
277,25	121,8333	125,6	9,6667	15,5
278,31	124,1667	126	9	14,6667
279,38	123,8333	129	10,1667	15,3333
280,44	121,8333	126,7	9,1667	14,8333
281,5	122,3333	126,7	9,1667	15,3333
282,56	127,3333	131	10,3333	14,1667
283,63	127,1667	122,2	11	14,3333
284,69	127,5	124,1	9,5	13,3333
285,75	126	122,2	8,3333	13,6667
286,81	127,6667	126,5	9	13,5
287,88	126,3333	126,6	9	15
288,94	127,6667	123,9	10,6667	14
290	126,1667	121,7	10,1667	15,1667
291,06	127,8333	124,1	9,5	14,3333
292,13	129	127,4	11	14,3333
293,19	126,5	124,8	10,1667	14,5
294,25	132	122,2	8,1667	14,5
295,31	128,5	122,9	10,8333	15,1667
296,38	127,6667	127,4	9,8333	15
297,44	126,8333	122,1	10,8333	14,1667
298,5	130,6667	124,1	8,3333	14,6667
299,56	130,1667	120,2	7,5	14,8333
300,63	132,5	120,5	6,5	14,6667
301,69	131,1667	120,8	10,3333	14,8333
302,75	132,1667	119,8	9,8333	15,6667
303,81	128,6667	119,5	9,3333	14,6667
304,88	127,1667	122,6	9,8333	15,5
305,94	122,6667	122,8	7,5	15,3333
307	118,3333	116,2	9,5	15,3333

308,06	120,1667	122	9,1667	15
309,13	124	117,6	8,1667	15,6667
310,19	124	120,4	9,1667	14,5
311,25	123,5	118,1	9,8333	15,8333
312,31	128,1667	120,9	8,3333	15,3333
313,38	131	117,5	8,8333	15
314,44	130,8333	119,5	9,1667	15,5
315,5	129,6667	123,4	7,5	14,6667
316,56	127,3333	115,8	9,5	14,8333
317,63	127,1667	116,9	9	14,3333
318,69	129,1667	116,7	7,3333	15
319,75	129,3333	113,9	9	14,6667
320,81	130,3333	114,7	10,5	15,1667
321,88	126,6667	113,3	8,1667	14,5
322,94	127,1667	112,4	7,3333	15
324	127,5	116,4	8,8333	15,3333
325,06	124,1667	108,5	8,8333	15,1667
326,13	124,8333	113,8	9,6667	16
327,19	124,1667	110,4	7,5	15,5
328,25	125,3333	114,2	9,1667	15,5
329,31	124,3333	114	6,8333	15,6667
330,38	124	112	8,3333	14,5
331,44	124,8333	112,6	7,8333	14,3333
332,5	125,5	107	8,8333	14,8333
333,56	124,5	109,6	8,6667	14,5
334,63	124,5	112,4	9,1667	14,8333
335,69	122,8333	109,6	10,6667	15,1667
336,75		111,3	9	15
337,81		117	7,6667	15,5
338,88		110,9	9,1667	14,6667
339,94		109,6	10,1667	14,8333
341		112,3	9,1667	15,3333
342,06		112,7	10,5	15,1667
343,13		112,8	9,1667	16
344,19		111,6	9,8333	15,1667
345,25		111,5	12,3333	14
346,31		107,5	12,1667	14,8333
347,38		108,8	10,8333	14,8333
348,44		111,6	10,1667	15
349,5		113,1	11,1667	16,1667
350,56		114,1	8,8333	14,8333
351,63		108,5	10,1667	15,6667
352,69		109	10	15,3333

353,75	106,3	9,3333	14,6667
354,81	104,9	10,6667	14,1667
355,88	104,7	10,6667	14,6667
356,94	105,7	11	14
358	108,3	10,5	14,6667
359,06	107,4	13,3333	15
360,13	104,4	13	14,1667
361,19	104,4	11,5	14,1667
362,25	104,8	12,1667	14
363,31	106,4	12,5	14,8333
364,38	102,5	13,3333	14,5
365,44	99,4	12,1667	14,5
366,5	103,2	12,3333	14,8333
367,56	105	12,8333	15,6667
368,63	107,3	12,1667	14,8333
369,69	108,8	12,6667	13,8333
370,75	105,1	13,8333	14
371,81	107	11,1667	14,6667
372,88	109,1	13	14,1667
373,94	107	14,3333	13,8333
375	109,9	14,1667	13,8333
376,06	106	12,5	14,8333
377,13	107	13,5	14,3333
378,19	108,4	12,3333	14,6667
379,25	107,4	13	14
380,31	108,2	13,6667	15,5
381,38	106,5	13,5	14,5
382,44	107,6	14,8333	13,6667
383,5	108,4	12,5	14
384,56	106,9	13,6667	13,6667
385,63	109	14,8333	13,5
386,69	104,7	14	13,1667
387,75	103,9	14,5	13,1667
388,81	105,9	13,3333	13,3333
389,88	105,4	15,3333	12,6667
390,94	104,9	13,6667	12,8333
392	104,8	12,3333	13
393,06	102	13,3333	13,1667
394,13	104,6	12,1667	14
395,19	100,9	14,5	13,5
396,25	99,3	12,8333	12,3333
397,31	97,6	12,1667	14
398,38	102	12,8333	12,5

399,44	101	11,8333	13,3333
400,5	103,3	12,8333	13
401,56	104	14,5	11,5
402,63	102,2	13,1667	12
403,69	101,4	12,5	11
404,75	102,7	12,8333	11,1667
405,81	99,6	14,3333	11,5
406,88	102,5	12,5	11
407,94	101,1	10,1667	10,6667
409	99,3	14,3333	10,1667
410,06	99,4	15,5	10,6667
411,13	98,1	13,6667	10,5
412,19	99	15,6667	10,3333
413,25	102,5	14,5	11,6667
414,31	95,6	14	11,5
415,38	98,3	13,1667	11,3333
416,44	100,1	14,6667	12,1667
417,5	96,7	13,1667	11,6667
418,56	96	13,1667	12
419,63	93,2	13,8333	12,5
420,69	94	13,1667	11,8333
421,75	95,7	12,1667	11,1667
422,81	93,6	16,3333	11,6667
423,88	96,6	14,6667	12,3333
424,94	95,8	15	12,6667
426	95,1	14,1667	13
427,06	97,7	16,3333	12
428,13	100,1	13,1667	12,5
429,19	92,5	12,3333	12,5
430,25	95,9	14,6667	12,6667
431,31	96,1	12	12,6667
432,38	96,1	12,5	12,5
433,44	96,9	14	12,6667
434,5	96,8	12,3333	12,8333
435,56	98,1	14,8333	12,5
436,63	94,1	14	12,8333
437,69	97,8	14	13,6667
438,75	92,3	14,3333	12,8333
439,81	91,4	13,6667	12,5
440,88	93,1	15	13,1667
441,94	91,3	14,3333	13,1667
443	92,3	12,8333	13,3333
444,06	94,3	14,5	12,5

445,13	89,1	14,5	13
446,19	90,2	15,1667	13
447,25	93,4	15	12,1667
448,31	91,5	15	12,3333
449,38	90	14,5	12,1667
450,44	96,1	13,6667	12,5
451,5	87,8	15,8333	13,1667
452,56	96,5	16,5	11,8333
453,63	90,3	17	13
454,69	91,5	16,5	10,8333
455,75	94,6	16,1667	10,1667
456,81	91,5	15,5	11,6667
457,88	91,3	17	11,5
458,94	90,6	16,3333	10,5
460	91,2	16	11,5
461,06	95,2	16,8333	12
462,13	86,7	15,5	12,3333
463,19	88,4	17,1667	13
464,25	88,1	17,5	12,5
465,31	85,8	18,8333	12
466,38	88,5	17,6667	11,6667
467,44	86,3	18,1667	12
468,5	89	17	13,1667
469,56	88,2	20,1667	12,1667
470,63	85,5	17,1667	12,5
471,69	87,6	17,8333	12,5
472,75	86,3	17,6667	11,8333
473,81	85,7	16,5	12,1667
474,88	86,7	18,6667	11,5
475,94	88,7	16,5	11,8333
477	90,3	19,5	12,1667
478,06	90,6	15,8333	12,5
479,13	87,3	18,1667	12,3333
480,19	83,3	19,1667	11,6667
481,25	89,8	18,8333	12,3333
482,31	83,7	17,8333	12,3333
483,38	82,6	19,8333	13
484,44	81,3	19,6667	12,3333
485,5	85	16,3333	12
486,56	84,4	17,5	12,3333
487,63	82	19,3333	12,6667
488,69	80,5	17,8333	12,1667
489,75	82	16,3333	11,8333

490,81	83,9	17,6667	13,1667
491,88	80,6	18	12,3333
492,94	80,1	19,1667	12,5
494	84,2	18,1667	12,8333
495,06	81,7	18,1667	12,8333
496,13	84,2	15,6667	11,6667
497,19	83,5	18,5	12,6667
498,25	79,8	18,3333	12,3333
499,31	83,9	20,6667	13,5
500,38	81,4	18,6667	13,5
501,44	82,5	19,3333	12,5
502,5	82,3	20,1667	12,8333
503,56	83,2	15,3333	13,8333
504,63	82,9	17,1667	12,6667
505,69	82,7	18,8333	12,5
506,75	86,8	16	12,1667
507,81	84,8	18,8333	12,3333
508,88	78,1	19	11,3333
509,94	84,1	18,5	12,3333
511	87,6	18,5	11,6667
512,06	80,8	19,5	12,3333
513,13	84,6	19	13,1667
514,19	80,3	19,5	12,1667
515,25	84,3	18,5	12,5
516,31	85,2	18,3333	12,5
517,38	83,2	20,5	12,5
518,44	86	17,8333	13,6667
519,5	85,7	18	13,1667
520,56	85	19,1667	12,5
521,63	85,9	17,3333	12,3333
522,69	84,6	19,8333	12,3333
523,75	90,7	18,5	11,6667
524,81	86	18,5	10
525,88	81,8	18,8333	10,8333
526,94	87,5	19,1667	11
528	83,9	18,8333	11,8333
529,06	81,1	19,8333	11,8333
530,13	80,9	20,5	11,6667
531,19	81,4	16,8333	11,1667
532,25	78,9	21,8333	11,8333
533,31	82,1	19,6667	11,5
534,38	85,5	19,8333	11,3333
535,44	80,8	21,3333	11,6667

536,5	81	19,6667	12,1667
537,56	81,7	19,6667	11,8333
538,63	79,5	20,5	13
539,69	80	20,5	12,5
540,75	80,2	20,1667	12,8333
541,81	81,2	19,6667	11,8333
542,88	83,8	21,5	13,1667
543,94	79,8	19,5	13,1667
545	83,9	21	12
546,06	79,2	19,8333	12,6667
547,13	83,6	19,6667	13,1667
548,19	80,2	20,8333	11,3333
549,25	79	20,1667	11
550,31	83,3	20,3333	12
551,38	76,1	21	12,1667
552,44	80,4	20,6667	13,5
553,5	79,1	21,6667	12,6667
554,56	84,9	19,5	13
555,63	82,3	20,3333	12
556,69	83,7	21,5	12,6667
557,75	85,9	20,6667	11,3333
558,81	77,5	22,3333	11,8333
559,88	81,3	19,6667	13,5
560,94	81,8	20,8333	12,3333
562	83,3	22,3333	12,1667
563,06	76,4	19	11,6667
564,13	77,3	21,5	10,6667
565,19	76,1	20	12,1667
566,25	76	20,5	11,6667
567,31	79,9	19,8333	12
568,38	75,3	19,8333	10,8333
569,44	78,4	20,1667	11,8333
570,5	75,8	21	11,1667
571,56	77,4	21,6667	11,6667
572,63	79,4	21,5	11
573,69	80,7	21	11,6667
574,75	80,8	22,1667	10,6667
575,81	78,1	22,5	12,3333
576,88	73,8	22,3333	11,1667
577,94	77,6	19,8333	11,1667
579	78,4	19,5	12,3333
580,06	77,2	20,1667	12,3333
581,13	76,9	21,8333	13

582,19	74	22,5	13
583,25	73,4	19,5	11,8333
584,31	74,1	20,5	13,6667
585,38	73,4	21,5	11,3333
586,44	76,7	22,5	11,5
587,5	74	23,1667	13
588,56	76	21	13
589,63	77	21,6667	13,1667
590,69	71,5	22,3333	14
591,75	70	23,1667	13,6667
592,81	69,8	22,1667	13,5
593,88	75,5	23,1667	12,6667
594,94	70,9	21,8333	13,8333
596	74,5	22,8333	13,8333
597,06	71,1	21,8333	12
598,13	71,8	22,8333	11,3333
599,19	71	24,5	12,1667
600,25	72,2	24,5	10,8333
601,31	75,9	23	11,5
602,38	74,8	25,5	10,5
603,44	71,8	27	11,1667
604,5	71,7	25,6667	12
605,56	69,9	28,1667	11,6667
606,63	73	26,5	11,5
607,69	69,5	30	11
608,75	72,9	26,6667	11,3333
609,81	71,4	28,1667	13,6667
610,88	72,9	25,6667	10,8333
611,94	69,3	28	11,1667
613	69,7	26,8333	12,1667
614,06	70,5	27,5	10,5
615,13	74,1	27,5	10,1667
616,19	72	28,1667	10,3333
617,25	69,9	25,8333	9,8333
618,31	75	27,1667	9,1667
619,38	73,4	30	9,3333
620,44	74,5	28	10,1667
621,5	74,6	28,3333	10,1667
622,56	74,8	29,6667	10,3333
623,63	69,7	28,1667	10,8333
624,69	72,2	29,6667	10,5
625,75	70,8	28,1667	10,3333
626,81	72,8	30	10

627,88	75,2	28,5	11,5
628,94	74,4	29	10,8333
630	71,2	31,1667	11,5
631,06	78,2	26,8333	10,6667
632,13	68,1	28,6667	12
633,19	73,3	27,5	11,3333
634,25	70,3	30,6667	11
635,31	74,1	29,5	12,1667
636,38	70,1	28,5	12
637,44	72,9	27,8333	11,5
638,5	73,1	28,8333	12
639,56	69,3	27,6667	11,1667
640,63	71,2	26,5	12,3333
641,69	69,3	27,6667	11
642,75	69,5	28,8333	12,1667
643,81	75,6	30,5	11,5
644,88	73	32,3333	12,8333
645,94	69,8	29,1667	12,8333
647	70,5	28,8333	12,1667
648,06	71,2	28,5	13,3333
649,13	67,7	27	12
650,19	70	27,8333	13,5
651,25	70,7	26,5	12,1667
652,31	71,3	28	12,8333
653,38	71,7	26	12,6667
654,44	69,7	28,8333	13
655,5	70,8	26,8333	13,6667
656,56	69,8	27,6667	12,3333
657,63	66,9	25,8333	12,8333
658,69	64,7	26,5	12
659,75	66,3	29,1667	12,5
660,81	64,8	27,3333	12,8333
661,88	67,4	29,6667	12,8333
662,94	65,9	29,5	13,1667
664	64,8	29,6667	12,6667
665,06	66,6	28,6667	12,5
666,13	64,1	29,8333	12,8333
667,19	66,7	29,6667	14,1667
668,25	68,7	29	12,8333
669,31	65,7	31	12,6667
670,38	66,7	29,5	13,6667
671,44	67,6	29,1667	12,3333
672,5	62,1	27,3333	12,8333

673,56	68,4	29,5	13,1667
674,63	65,4	29,5	11,8333
675,69	64,4	30	10,5
676,75	63,9	28,3333	11
677,81	63	29,8333	10,1667
678,88	63,8	30,1667	12
679,94	62,6	31,3333	11
681	66,2	30	12,3333
682,06	66,7	29,6667	13
683,13	67,2	31,3333	12,1667
684,19	65,9	27,6667	13,1667
685,25	65,6	28,5	12,3333
686,31	64,9	28,5	13,1667
687,38	60,7	28,6667	13,5
688,44	63,1	28,8333	13
689,5	68,1	31,1667	13,6667
690,56	65	30,3333	12,5
691,63	67,5	31,5	12
692,69	64,3	30,1667	12,8333
693,75	63,2	28,8333	11,8333
694,81	63,5	30,3333	12,6667
695,88	60,3	29,5	13,5
696,94	65,1	30,8333	12,6667
698	63,6	29,8333	13,5
699,06	62,3	31,1667	13
700,13	64,4	31,5	12,8333
701,19	58,3	30,6667	13
702,25	65,1	30,5	13,3333
703,31	67,6	31,8333	15
704,38	63,9	30,3333	12,8333
705,44	65	29,1667	13,3333
706,5	63,3	31	14
707,56	63,2	28	13,8333
708,63	64	29	13,8333
709,69	67,9	30,6667	13,5
710,75	64,8	29,1667	13,5
711,81	62,1	31,6667	13,3333
712,88	66,1	29,8333	12
713,94	62,8	31,1667	12,8333
715	65,8	30,5	12,5
716,06	62,7	31	12,8333
717,13	67,8	30,8333	13
718,19	60,1	31,1667	14,5

719,25	63,1	31,1667	12,6667
720,31	64,4	31,1667	14,1667
721,38	65,3	30	11,6667
722,44	64,2	31,6667	13,5
723,5	62	30,6667	11
724,56	63,6	32,3333	11,1667
725,63	60	32,3333	11,5
726,69	62,9	32,5	9,8333
727,75	59,5	32,5	10,3333
728,81	61,6	31,1667	11,8333
729,88	60,6	32,8333	11,5
730,94	59,4	36,6667	10,5
732	59,3	31,8333	11,6667
733,06	61	34,6667	11,6667
734,13	62,6	34,8333	11
735,19	62,6	31	10,3333
736,25	62,8	33,3333	10,6667
737,31	63,2	34	11,6667
738,38	61,4	34,6667	11,3333
739,44	64,4	34,3333	11,3333
740,5	61,1	34,6667	14,1667
741,56	63,7	36,3333	12,5
742,63	58,5	34,1667	12,6667
743,69	62,1	35,6667	13
744,75	61,4	33,5	13,1667
745,81	63,4	32,6667	12,6667
746,88	61	35,3333	12,6667
747,94	61,3	35,5	12
749	60,5	34,6667	13,3333
750,06	62,3	36,1667	13,3333
751,13	61,4	36	14,1667
752,19	59,6	35,8333	13,1667
753,25	59,4	36	13,6667
754,31	61,6	37,5	11,8333
755,38	62,3	36,5	12,6667
756,44	65,2	37,3333	13,6667
757,5	59,5	37,5	15,1667
758,56	63,4	34,5	12,8333
759,63	65,2	35	14
760,69	65,1	36,1667	11,8333
761,75	61,7	36,3333	10,8333
762,81	60,6	35,5	10,1667
763,88	62,1	35,1667	9,8333

764,94	61,4	35,1667	11
766	60,3	38	11,1667
767,06	59	38,5	11,3333
768,13	60,9	34,6667	11,1667
769,19	55,8	35	12,3333
770,25	58,4	36,5	12,1667
771,31	61,4	36,3333	10,5
772,38	68,4	37,5	10,5
773,44	56,7	37	11,1667
774,5	63,5	36,3333	10,8333
775,56	58,9	36,5	12,3333
776,63	65	35,1667	11,1667
777,69	64,9	36,3333	12
778,75	65,3	35,5	11,8333
779,81	60,7	37,5	12,1667
780,88	61,5	37	10,8333
781,94	66,2	35,5	10,5
783	59,8	38	13
784,06	60,9	35,8333	13,5
785,13	61,8	38,1667	10,8333
786,19	59,2	36,6667	11,5
787,25	61,8	38,1667	10,5
788,31	61,9	37,8333	10,3333
789,38	59,5	37,8333	10,5
790,44	61,8	39	11,8333
791,5	60,9	38,6667	10,3333
792,56	60,7	42,3333	12,1667
793,63	63,7	40,3333	12,3333
794,69	66,6	37,6667	11,8333
795,75	58,9	40,5	12,3333
796,81	64,8	40	11,6667
797,88	60,6	39,3333	11,6667
798,94	58,7	39,5	13,1667
800	59,6	37	12,6667
801,06	57,4	41	12,8333
802,13	61,6	40,5	13,5
803,19	61,6	40,8333	12,3333
804,25	63	41,1667	12,8333
805,31	60,5	41,3333	13,3333
806,38	59,9	39,5	14
807,44	64,3	42	12,5
808,5	60,1	42,6667	14,1667
809,56	57,3	41,6667	13

810,63	62,9	42,1667	13,6667
811,69	61	43,1667	13,3333
812,75	59,7	40,8333	13,3333
813,81	59,7	40,6667	12
814,88	64,3	41,5	14,3333
815,94	60,6	42,1667	14
817	58	41,3333	12,5
818,06	60,2	41,5	13,8333
819,13	59,7	42,8333	14,5
820,19	57,3	44	12,6667
821,25	61,8	42,6667	14,1667
822,31	60,4	41,1667	13,5
823,38	58,8	43,5	13,8333
824,44	62,4	43	14
825,5	65,2	43,5	13,1667
826,56	60,7	40,8333	13,8333
827,63	61,3	42,5	12,1667
828,69	60,1	43,8333	10,8333
829,75	64,5	44	12,5
830,81	62,2	42,8333	13,6667
831,88	58,1	43,1667	12,5
832,94	60,2	43	12,6667
834	62	42,5	12
835,06	60,6	43,3333	14
836,13	61,1	45,5	12,1667
837,19	60,1	46,6667	14
838,25	59,9	46	11,1667
839,31	59,6	44,6667	12,1667
840,38	58	45,5	11,1667
841,44	58,6	43,6667	12,3333
842,5	60,2	46,5	12,5
843,56	61,3	43,6667	12,6667
844,63	62,7	44,3333	13
845,69	62,2	45,8333	14,5
846,75	63,8	45,3333	14,3333
847,81	61,5	45,3333	13,8333
848,88	61,2	44,5	12,1667
849,94	58,2	45,6667	13,1667
851	60,6	44,6667	13
852,06	58,6	46	13,6667
853,13	64	44,8333	13,1667
854,19	62,1	44,5	12,5
855,25	52,5	44	11,3333

856,31	65,6	45,3333	13,5
857,38	98,9	42,6667	11,3333
858,44	107,9	46,1667	11,8333
859,5	108,2	48,3333	11,6667
860,56	111,4	46,8333	13,1667
861,63	111,6	46,6667	11,6667
862,69	113,2	46,6667	11,3333
863,75	119,4	47,6667	12
864,81	117,6	45,6667	13,5
865,88	120,8	47,1667	13,3333
866,94	122,6	47,8333	14,3333
868	114,9	45,5	13,1667
869,06	119,1	46,1667	12,6667
870,13	116,5	47,5	14,1667
871,19	120,3	47,8333	14,8333
872,25	119,4	47,6667	13,5
873,31	117,1	48,5	13,3333
874,38	117,2	47,1667	14,5
875,44	117,8	47,5	13,1667
876,5	113,1	49	11,6667
877,56	117,6	48,1667	12,8333
878,63	118,8	49,1667	14,3333
879,69	120,1	48,5	14,8333
880,75	116,4	48,1667	12,6667
881,81	112,6	47	13,3333
882,88	116,7	46,8333	13,1667
883,94	119,4	50,6667	13,8333
885	119,8	48,5	13,3333
886,06	117,7	49,3333	13
887,13	113,1	50	12,3333
888,19	113,8	50	14,6667
889,25	114,4	44,8333	14,5
890,31	113,2	64,1667	13,5
891,38	115,1	77	14
892,44	115,7	81,3333	13,8333
893,5	112	86,8333	13,8333
894,56	113,5	94,3333	9
895,63	110,9	104,5	11,8333
896,69	112,6	113,5	15,6667
897,75	117,4	124,8333	16,5
898,81	107,8	130,3333	21,1667
899,88	113,9	135	23
900,95	116,3	144,3333	24,3333

902,02	114,1	145,6667	27
903,08	115,4	150,6667	29,5
904,14	113,2	153,5	29,3333
905,2	114,1	155,6667	31,3333
906,27	115,2	157,5	32,5
907,33	113,7	158,6667	34
908,39	116	163,1667	38
909,45	117,5	162,8333	40,6667
910,52	116,6	164,1667	43,1667
911,58	118,3	165,5	45
912,64	114,4	165,5	47,8333
913,7	117	168,6667	48,8333
914,77	111,6	161,8333	53
915,83	114,5	166,6667	53,6667
916,89	110,1	166,1667	55,5
917,95	116,8	167,1667	58
919,02	112,4	167,1667	59,5
920,08	116,9	163,5	61
921,14	115	163,1667	60,3333
922,2	108,9	166,6667	59
923,27	109,1	160,6667	59,5
924,33	111	158	58,6667
925,39	113,5	156,1667	58,6667
926,45	112,1	153,8333	57,8333
927,52	112,7	155,8333	57,1667
928,58	111,8	152,3333	56,1667
929,64	111,2	152,6667	54,8333
930,7	107,6	153,3333	55
931,77	109,2	152,5	55
932,83	111,6	157,6667	56,3333
933,89	114,2	154,5	54
934,95	109,8	154,3333	54,3333
936,02	113	149,5	52,3333
937,08	107	153,6667	52,5
938,14	113,9	155	51,3333
939,2	106,8	156,3333	48,8333
940,27	113	153,6667	47,8333
941,33	115,2	154,5	48,1667
942,39	112,3	150,3333	47,5
943,45	106,3	153,5	50,3333
944,52	110	153	49
945,58	109,4	152	46,5
946,64	105,5	150,3333	44

947,7	108,2	155,3333	44,5
948,77	104,2	152,1667	46
949,83	111	154,5	46,3333
950,89	109	155,3333	46,5
951,95	109,2	155,6667	44,5
953,02	107,5	155,6667	43,6667
954,08	109,1	155,3333	44,3333
955,14	109,7	152,1667	42,1667
956,2	108,9	150,6667	41,6667
957,27	107,8	155,5	43,5
958,33	108	155,8333	42,1667
959,39	109,7	158,8333	40,6667
960,45	107	154,6667	40,6667
961,52	112,1	154,6667	40,6667
962,58	113,7	155,3333	40,5
963,64	107,3	155,3333	40,5
964,7	109,4	158,3333	41,3333
965,77	108,4	153,6667	40
966,83		154,5	40,8333
967,89		155	40,5
968,95		150	39,3333
970,02		152,6667	37,8333
971,08		150,3333	37,5
972,14		151,6667	39,1667
973,2		155	40,3333
974,27		153,8333	37,3333
975,33		153,1667	38,8333
976,39		154,5	36,8333
977,45		150,3333	36
978,52		155,5	36,5
979,58		151,6667	37,3333
980,64		156,8333	37,5
981,7		155,1667	37,3333
982,77		154,3333	37,1667
983,83		153	35,5
984,89		151,6667	36,6667
985,95		153,6667	36,8333
987,02		153,5	35,6667
988,08		151,6667	37,3333
989,14		153,3333	37,5
990,2		154	37,8333
991,27		152,8333	36,6667
992,33		152,6667	36

993,39	150,6667	35,5
994,45	147,8333	36,6667
995,52	151,3333	35,5
996,58	147,6667	36,5
997,64	145,6667	37,1667
998,7	150,3333	37,3333
999,77	149,5	37,8333
1000,83	149,5	36,3333
1001,89	148,5	35,5
1002,95	150,8333	38
1004,02	150,6667	36,5
1005,08	148,1667	36,3333
1006,14	148,5	37,5
1007,2	149,8333	37
1008,27	149,8333	36,6667
1009,33	152,5	36
1010,39	150,5	35,1667
1011,45	147,8333	37,5
1012,52	147,8333	36,1667
1013,58	146	34,1667
1014,64	147,5	34,5
1015,7	146,6667	35,5
1016,77	150	34,6667
1017,83	148	32,5
1018,89	150,8333	35,5
1019,95	145,6667	33,6667
1021,02	146,6667	34,5
1022,08	151,3333	33,8333
1023,14	149,6667	34,8333
1024,2	145,3333	36
1025,27	148,1667	36,5
1026,33	144,8333	33,8333
1027,39	148,1667	34,5
1028,45	147	34,3333
1029,52	149	34,8333
1030,58	145	34,1667
1031,64	145,8333	34,1667
1032,7	145,5	34,6667
1033,77	149,1667	34,5
1034,83	147,6667	34,8333
1035,89	146,5	33
1036,95	148,1667	33,3333
1038,02	147	33,6667

1039,08	147,1667	32,5
1040,14	146,6667	32,6667
1041,2	147,5	32,5
1042,27	147,8333	33,1667
1043,33	146	34
1044,39	147,5	35
1045,45	148	34,6667
1046,52	149,5	33,5
1047,58	147,1667	31,8333
1048,64	148,5	33,3333
1049,7	146,6667	33,5
1050,77	148,8333	33,8333
1051,83	147,3333	33,3333
1052,89	148	32,5
1053,95	146	33
1055,02	144,8333	32,8333
1056,08	146,3333	32,5
1057,14	147,1667	32,1667
1058,2	148,1667	32,5
1059,27	144,5	32,1667
1060,33	144,6667	32,3333
1061,39	141,8333	32,8333
1062,45	142,3333	33
1063,52	142	32,6667
1064,58	145,5	32,8333
1065,64	144,6667	33,6667
1066,7	142,8333	32,3333
1067,77	141,8333	33
1068,83	143,1667	32,6667
1069,89	142,3333	33,8333

TABLE S6. Effect of 15 min IL Incubation on Histamine Stimulated Increase in $[Ca^{2+}]_i$ – Peak $[Ca^{2+}]_i$

Alone	After IL4	After IL5	After IL13
414,66	22	39,11	15,56
132,78	70,1	111,8	17
279,9	15,45	6,47	15,75
314,4	104,6	381,5	0,55
299,16	199,8	123,84	49,17

TABLE S7. Effect of 24h IL Incubation on Histamine Stimulated Increase in $[Ca^{2+}]_i$ – Time Traces

Time	Histamine	IL4 + Histamine	IL5 + Histamine	IL13 + Histamine
0,75	145,6667	9	6,4286	10,6667
1,83	146	9,3333	5,8571	10,3333
2,91	141,5	8,6667	5,5714	10,2222
3,98	142	10,1667	4,8571	10,3333
5,06	145,6667	9	6,7143	9,3333
6,14	143,8333	9,5	6,7143	10,2222
7,22	143	8,6667	6,4286	10
8,3	145,3333	9,1667	4,8571	10,2222
9,38	144,3333	10,5	5,5714	10,8889
10,45	146,1667	10,5	6,1429	10,4444
11,53	147,1667	9,1667	6	10,4444
12,61	145,6667	10,5	6,7143	9,8889
13,69	146,1667	10,3333	6,2857	10,8889
14,77	144,6667	11	0	10,1111
15,84	144	4	24,5714	6,3333
16,92	144	2,6667	53,7143	14,8889
18	145,5	17,5	80,8571	22,7778
19,08	146,8333	39,1667	91,7143	28,6667
20,16	111,8333	63	107,1429	33,3333
21,23	149,1667	84	114,8571	35,7778
22,31	141,8333	115	125,8571	38,4444
23,39	148	146,1667	130,4286	40,8889
24,47	163,3333	165,5	130	43,7778
25,55	203,6667	183,1667	130,2857	44,6667
26,63	247	192,8333	127,2857	46,6667
27,7	284	190,6667	124,5714	48,6667
28,77	311,5	196,3333	124,8571	52
29,84	329,6667	197,5	121,1429	54,8889
30,91	346,1667	199,6667	112,2857	56
31,98	354,8333	195,8333	112,4286	57,4444
33,06	372,8333	208,5	114,7143	58,6667
34,14	368,8333	210,1667	111,4286	60,6667
35,22	378,6667	203,8333	109,8571	59,8889
36,3	382,5	199,1667	108,5714	61,2222
37,38	382,8333	201,6667	108,4286	59,4444
38,45	388,8333	208,1667	108,5714	61,4444
39,53	396,1667	206,5	107	58
40,61	393,5	210,3333	105,7143	59,2222
41,69	396,8333	204,8333	104,7143	57,7778
42,77	400,3333	203,6667	102,8571	56,5556

43,84	402,3333	205	101	58
44,92	390,6667	196,5	101	58
46	394	195	96,1429	57,4444
47,08	394,8333	196,5	95,4286	55,2222
48,16	392,6667	198,3333	94,7143	57,1111
49,23	397,3333	204	90	55,5556
50,31	395	205,1667	91,4286	56,4444
51,39	394,3333	205,8333	90,1429	57,1111
52,47	396,6667	206,1667	88,1429	55,5556
53,55	400	208,8333	90,2857	55,4444
54,61	387,1667	206,8333	90,8571	56,5556
55,69	393,3333	211	88,8571	55,4444
56,75	389,8333	217,5	86,7143	55,4444
57,81	392,8333	211,3333	89,2857	55,6667
58,88	385,3333	217	86	55,8889
59,94	387,1667	221,3333	87,2857	55
61	385	221,8333	85	54,8889
62,06	387,6667	222,3333	84	56,3333
63,13	383	219	83,7143	56
64,19	384,5	220,1667	83,7143	55,5556
65,25	381,1667	225,8333	80,5714	55,7778
66,31	381,5	221,3333	78,1429	57,1111
67,38	380,1667	219,6667	77,4286	56,2222
68,44	380,3333	218,8333	83	56,1111
69,5	373,1667	221,3333	83,2857	56,7778
70,56	376,5	220,3333	81,4286	56,1111
71,64	377,5	223,5	78,4286	56,2222
72,7	370,8333	213,8333	77	55,7778
73,77	368,6667	216,8333	76,8571	56,3333
74,84	369,8333	217,1667	79,2857	56
75,91	366,8333	218,6667	78,4286	55,4444
76,97	362	216	79,7143	55,7778
78,03	359	216,8333	80	55
79,11	359,5	216,6667	72,4286	54,3333
80,17	351,6667	216,6667	75,5714	55,1111
81,23	352,8333	222,5	75,4286	54,7778
82,3	352	221,3333	77,1429	54,5556
83,36	357,3333	224,3333	77	54
84,42	352,8333	229,5	74,2857	53,3333
85,48	346,5	225,8333	76,4286	54,4444
86,55	342,8333	222,8333	73,2857	52,7778
87,63	337,1667	224,3333	76	52,7778
88,69	336,6667	224,6667	72,4286	51,3333

89,75	343,8333	223,5	75,4286	52,2222
90,81	330,5	220	77,4286	52,3333
91,88	336,3333	219,5	72,7143	51,2222
92,94	330,5	216,5	70,8571	50,7778
94,02	329,8333	220,5	74,5714	50,4444
95,08	333,6667	222,3333	71,8571	51,4444
96,14	332,8333	216	71,4286	50,3333
97,2	326,8333	216,1667	71,2857	50,7778
98,27	317,5	214,5	72,4286	51
99,34	325,5	211,1667	74,2857	50,2222
100,41	329,3333	220,8333	72,4286	49,7778
101,47	318,8333	225	76,5714	51,1111
102,53	325,6667	226,1667	71,8571	50,6667
103,59	318	221	71,8571	51
104,66	319,1667	215,8333	74	50,1111
105,72	314,3333	227	71,8571	49,3333
106,78	314,1667	214,1667	74,2857	49,7778
107,84	313,6667	217,6667	70,7143	48,7778
108,91	310,6667	219,6667	70,4286	48,5556
109,97	311,3333	226,6667	75,4286	48,8889
111,03	308	222,5	71,7143	49,2222
112,09	310,1667	220	71,8571	48,5556
113,16	311,3333	217,5	70,2857	48,7778
114,22	310,3333	222,5	70,1429	48,3333
115,3	307	218,5	68	47,3333
116,36	310,3333	215,1667	65,5714	48,8889
117,42	307,8333	212,5	67	47,8889
118,48	304,5	211,5	69,2857	48
119,55	307,3333	212,3333	66,8571	47,4444
120,61	305	213	67,8571	48,5556
121,67	302,5	220	68,5714	49,4444

TABLE S8. Effect of 24h IL Incubation on Histamine Stimulated Increase in $[Ca^{2+}]_i$ – Peak $[Ca^{2+}]_i$

Alone	After IL4	After IL5	After IL13
480	18,43	16,83	0,77
220,57		177,71	65,66
187,44	304,17	82,79	20,09
121,56	43,875	337,8	123,875
250,43	227,875	34,29	181,875

TABLE S9. Effect of Histamine Dimaleate (HD) Alone and After Cytokine Pretreatment in Peak $[Ca^{2+}]_i$

HD Alone	After IL4	After IL5	After IL13
304,385	509,25	42,13	105,42
242,6741667	58,85714286	35,69230769	131,8035714
466,9722222	157,4230769	17,56	42,48148148
360,4113757	203,5714286	67,67897727	298,5666667
368,3931667	170,62	100,16	81,17

TABLE S10. Effect of Cytokines Alone of After Histamine Dimaleate (HD) Alone Pretreatment in Peak $[Ca^{2+}]_i$

IL4	IL4 after HD	IL5	IL5 after HD	IL13	IL13 after HD
115,2	103,12	214,08	93,25	356,145	110,28
148,3571429	217,7333333	51,76923077	57,23333333	466,4642857	248,875
475,9230769	170,3	102,28	57,875	115	78,88888889
210,8571429	91,11111111	422,9378157	644,5714286	298,2333333	158,5
192,315	155,7825	119,155	58,215	487,811	123,775

TABLE S11. Extracellular Calcium Stores

IL4	IL4 no Ca^{2+}	Ca^{2+}	IL5	IL5 no Ca^{2+}	Ca^{2+}
236,6633333	82,645	186,715	231,395	254,83	237,61
721,7275	334,695	668,385	464,675	64,24	
446,24	93,125	777,33			
415,825	363,27	391,925	565,42	504,49	321,945
545,245	217,43	397,505			

IL13	IL13 no Ca2+	Ca2+	HD	HD no Ca2+	Ca2+
592,73	61,96	340,5	515,76	47,067	1184,8
476,09	61,64	395,82	457,735	297,375	610,67
171,97	152,55	646,55	239,005	36,62	214,38
819	22,3	142,97	591,21	467	882,29
165,845	635,32	769,77	420,09	56,81	192,75

TABLE S12. Intracellular Calcium Stores

IL4	IL4 after thapsigargin	IL5	IL5 after thapsigargin	IL13	IL13 after thapsigargin	HD	HD after thapsigargin
236,66333	150,635	231,395	137,67	592,73	76,17	515,76	116,67
721,7275	413,945	464,675	26,37	476,09	62,72	457,735	184
446,24	499,015			171,97	225,7	239,005	69,97
415,825	423,31	565,42	65,74	819	103,825	591,21	31,66
545,245	371,6			165,845	65,57	420,09	440

TABLE S13. Change in peak $[Ca^{2+}]_i$ After Treatment with IL15, Histamine Dimaleate (HD) and β-ARK1

HD	IL5 + HD	β-ARK1 + HD	β-ARK1+ IL5 + HD
457,735	78,96	142,59	391,17
239,005	442,54	280,83	423,04
89,77	54,79	222,74	290,96
591,21	321,29	835,94	1017,39
420,09	204,415	329,06	482,56
153,7533333	61,705		763,18
631,055	26,56	114,95	39,46
321,86	128	84,5	396,67

TABLE S14. Change in peak $[Ca^{2+}]_i$ After Treatment with IL13, Histamine Dimaleate (HD) and β-ARK1

HD	IL13 + HD	β-ARK1 + HD	β-ARK1 + IL13 + HD
196,76	33,175	804,89	73,56
744,7725	13,55	497,66	427,4
472,61	73,28	518,645	95,87
634,165	126,45	479,37	210,04

TABLE S15. Effect of IL4 and IL13 on each other's increase in $[Ca^{2+}]_i$

IL4	IL4 after IL13	IL13	IL13 after IL4	IL4+IL13
198,83	92,345	440,02	667,095	279,515
688,5	135,18	587,481	26,81	335,125
486,6	263,615	394,9	272,8	217,91
271,5	118,62	802,95	543,885	300,9
201,44667		449,265	367,06667	
514,51	100,025	285,19	116,15	116,59
262,5	263,6	264,1	553,5625	115,655
478,835	156,095	464,175	367,085	145,44

TABLE S16. Goblet Cell Secretion Mediated by Cytokines

Basal	IL4	IL5	IL13
1	1,757	0,863	1,796
1	9,144	4,573	19,087
1	3,411	1,564	0,722
1	1,301	2,045	1,876
1	0,794	1,163	2,272

TABLE S17. Histamine-Induced Goblet Cell Secretion

Basal	Hist	IL4 + Hist	IL5 + Hist	IL13 + Hist
1	1,629	0,414	1,153	3,422
1	2,702	0,433	4,255	16,184
1	1,813	2,886	1,062	1,708
1	1,454	1,663	2,018	2,445
1	3,064	5,848	2,409	1,003

TABLE S18. Goblet Cell Proliferation

Basal	IL4	IL5	IL13	Histamine	EGF
1,00	1,150883904	1,20113951	1,333899767	0,903715843	
1,00	0,987030737	1,024888821	1,031095064	0,785472358	1,091661198
1,00	3,650180178	3,615948941	4,141169151	1,188836948	3,897839444
1,00	1,55646814	1,434325969	1,897382325	1,007364713	1,323294909
1,00	3,62578922	5,468345046	5,2771859	0,402975522	5,211280948
1,00	1,36658102	1,492413272	2,080583208	0,331620078	1,779785095
1,00	1,41168122	1,129884811	1,138687517	1,222235196	1,474456131
